# Quantification of the porosity in template-based ordered porous Ag electrodes and its effect on electrochemical CO_2_ reduction†

**DOI:** 10.1039/d5re00068h

**Published:** 2025-06-25

**Authors:** Maaike E. T. Vink-van Ittersum, Erik Betz-Güttner, Eric Hellebrand, Claudia J. Keijzer, Matt L. J. Peerlings, Peter Ngene, Petra E. de Jongh

**Affiliations:** a Materials Chemistry & Catalysis, Debye Institute for Nanomaterials Science, Utrecht University Universiteitsweg 99 3584CG Utrecht The Netherlands p.e.dejongh@uu.nl; b Department of Earth Sciences, Utrecht University The Netherlands

## Abstract

The electrochemical reduction of CO_2_ combined with efficient CO_2_ capture is a promising approach to close the carbon cycle. We studied the effect of pore size on the activity and selectivity of porous Ag electrodes using template-based electrodes as model catalysts. Using polymer spheres with sizes between 115 nm and 372 nm as templates, ordered porous Ag catalysts with different pore diameters were obtained. These well-defined model systems allowed us to understand the effect of pore size on CO and H_2_ production. At the most cathodic potential, around −1.05 V, up to 4 times more CO than H_2_ was formed. The intrinsic CO production depends on the pore size, as it increases when changing the pore diameters from ∼100 nm to ∼300 nm. At pore diameters above ∼300 nm, the pore size does not affect the intrinsic CO production anymore. For the first time, FIB-SEM was used to quantitatively analyse the porosity of the electrodes and correlate it with trends in intrinsic activity. The catalyst with a pore diameter of ∼200 nm had the highest tortuosity of 2.41, which led to an increased CO production. The catalysts with a pore diameter of ∼200 nm and smaller have pore networks that are twice as long as the pore network of catalysts with ∼400 nm pores. This leads to an additional potential drop, which lowers the effective driving force for the electrochemical reaction. Disentanglement of these different factors is important for rational design of porous CO_2_ reduction catalysts.

## Abstract 2

Pore size effects in the e-CO_2_RR were studied using templated Ag. The intrinsic activity to CO increases upon increasing pore size. Quantification of the porosity showed that this is due to a higher tortuosity and additional potential drops.

## Graphical abstract

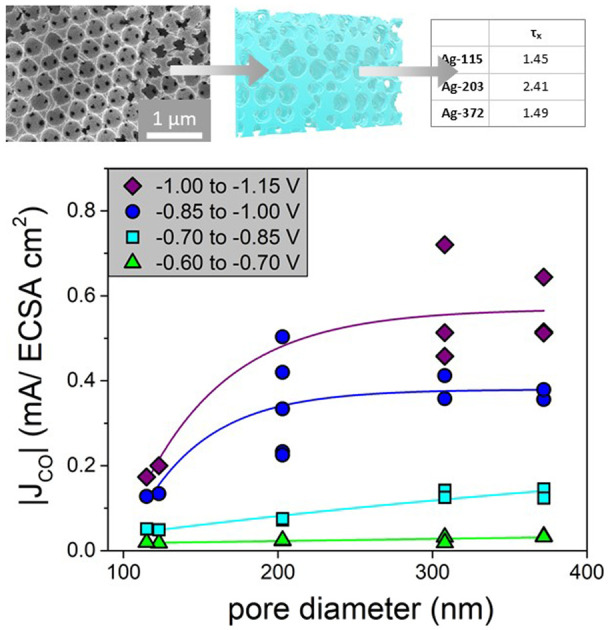

## Introduction

The capture of CO_2_ from the air followed by electrochemical CO_2_ reduction is a promising approach to store intermittent electricity from renewable sources in chemical bonds and to close the carbon cycle. Next to efficient CO_2_ capture driven by renewable energy, good catalysts converting CO_2_ at high current densities (>100 mA cm^−2^) have to be designed for the large-scale application of this conversion.^1^ Hori's pioneering work classified the transition metals into formate (Sn and Bi), CO (Ag, Au, and Zn), H_2_ (Pt and Pd), and C_2+_-product (Cu) producing catalysts.^2–6^ Due to its clear selectivity, its high stability compared to Cu and the fact that is easy to prepare, Ag has been the metal of choice for these fundamental studies.

The use of porous metal electrodes is promising to achieve high current densities because of their high specific surface areas. Porous structures might also have disadvantages, such as diffusion limitations of reactants and products.^7–9^ Also, their often complex structure makes it hard to disentangle the effect of different parameters. Hence, porous model catalysts are very useful for obtaining the fundamental understanding of the processes taking place in the porous electrodes.

In this work, we focus on porous model catalysts synthesized using ordered templates. Self-assembled polystyrene (PS) or poly(methyl methacrylate) (PMMA) spheres have been used to synthesize materials ranging from metal oxides such as Al_2_O_3_, TiO_2_, NiO, Co_3_O_4_, Fe_2_O_3_ and ZnO to metal alloys such as Ni_*x*_Co_*x*−1_, Mn_3_Co_7_ and AgAu.^10–15^ In the case of CO_2_ reduction, porous Ag catalysts were prepared through Ag deposition in the voids of a PS template on Au-coated glass slides. For these templated Ag electrodes, the mesostructure, particularly the catalyst thickness, had a significant impact on the selectivity. Thicker catalyst layers improve CO production and suppress H_2_ formation.^9^ Computational models predict that not only the thickness of the porous catalyst layer but also the pore diameter influences the catalytic performance. Smaller pores should lead to more CO production.^8^ However, to our knowledge, no systematic studies have been reported on the effect of the pore diameter in porous Ag. In particular, potential diffusion limitations in well-defined model systems are interesting. To date, the electrodes have always been prepared on very flat substrates (ITO and Au-coated glass).^15,16^ Meanwhile, they have not been explored yet on commonly used microstructured substrates, such as carbon paper for gas diffusion electrodes. Therefore, in this work, we prepared porous Ag electrodes from PMMA sphere templates on carbon paper and we analysed the effect of pore size and structure on the catalytic performance. The results show that the intrinsic activity depends on the pore size and that smaller pores lead to a lower intrinsic activity. We used FIB-SEM to quantitatively explain these results. A higher tortuosity and additional potential drops are the cause for a lower intrinsic activity for smaller pore sizes.

## Experimental

### Synthesis

**Fig. 1:**
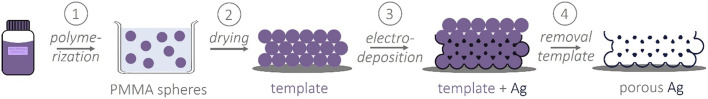
Schematic representation of the synthesis of template-based porous Ag by electrodeposition of Ag on a PMMA sphere template, involving 1) the synthesis of PMMA spheres; 2) the formation of a template on a substrate; 3) electrodeposition of Ag and 4) removal of the template. Adapted from Vink-van Ittersum *et al.*^17^

The porous catalyst synthesis consisted of four steps as described in the previous work of this group.^17^ The four steps are the synthesis of PMMA spheres, the formation of a template, the electrodeposition of Ag, and finally, the removal of the template (Fig. 1). The PMMA spheres were made based on a polymerization reaction of methyl methacrylate (MMA, Sigma Aldrich, >99%) in MilliQ water described by van den Reijen *et al.*^10^ following the work of Schroden *et al.*^18^ and Zou *et al.*^19^ By varying the MMA concentration between 0.5 M and 1.9 M, the stirring rate between 450 rpm and 600 rpm and the reaction temperature either 70 °C or 80 °C, six batches of PMMA spheres with different diameters were prepared. The reaction conditions for each PMMA batch are listed in Table 1. The PMMA sphere suspensions were (diluted and) dried on carbon paper (Toray TGP-H-060) on a heating plate at 80 °C to form the PMMA electrode.

**Table 1:** Reaction conditions (the monomer concentration, stirring rate and heating rate) during the synthesis of PMMA templates for the samples PMMA-87, PMMA-115, PMMA-123, PMMA-203, PMMA-308 and PMMA-372 and their average sphere diameters and polydispersity index

Sample	[Monomer] (M)	Stirring rate (rpm)	Heating *T* (°C)	Average diameter (nm)	Polydispersity index
PMMA-87	0.5	500	80	87	0.0036
PMMA-115	0.5	450	70	115	0.0052
PMMA-123	0.5	600	80	123	0.0027
PMMA-203	0.9	450	70	203	0.0024
PMMA-308	1.9	450	70	308	0.0057
PMMA-372	1.9	600	80	372	0.0031

The silver structure, which is an inverse of the template, was made by electrodeposition from a solution of 0.05 M AgNO_3_ (Alfa Aesar, 99.9+%), 0.5 M NH_4_OH (Emsure, 28–30%), 1.0 M NaNO_3_ (Alfa Aesar, 99.0%) and 0.01 M EDTA (Sigma Aldrich, 98–103% and Acros Organics, 99%) stirred at 500 rpm. A three electrode set-up was used, consisting of a Pt anode, a 3 M Ag/AgCl reference electrode and a glassy carbon disc (SIGRADUR K disc) with the PMMA electrode as the cathode. A potential of −0.2 V *vs.* Ag/AgCl was applied until a total charge of 2 C cm^−2^ had passed, which corresponds to a loading of 2.2 mg Ag cm^−2^. Based on the previous work in this group,^17^ we assumed that only Ag deposition was taking place at this potential. Thereafter, the electrode was rinsed with MilliQ water and left to dry. Finally, the PMMA template was removed by soaking the electrode in approximately 10 mL acetone for at least 1 h. Subsequent drying in air gave the final electrode.

### Characterization

To characterize the catalysts, several techniques were used. Scanning electron microscopy (SEM) was used to image the structure of the catalysts and to determine the pore size distribution. Electron images were acquired on a Thermo Fisher Scientific (TFS) Helios G3 UC operated at 2 kV and 50 pA. The particle sizes were counted using ImageJ^20^ to determine both the particle size distribution and polydispersity index (PDI) of the particle sizes. For each PMMA sample, the diameter of at least 400 particles was determined. The polydispersity index is defined asPDI = *σ*^2^/*μ*^2^where *σ* is the standard deviation in the particle size distribution and *μ* the average sphere size. If this value is smaller than 0.04, a monodisperse particle size distribution is obtained.^21^

Focused-ion beam scanning electron microscopy (FIB-SEM) images were obtained using the same TFS Helios G3 UC SEM. For the ion milling, the stage was tilted to 52° with the ion beam positioned perpendicular to the surface. A Ga-ion source operating at 30 kV and 0.40 nA was used as the ion beam and Auto Slice & View 4 software of FEI was used to automatically slice through the sample. For the Ag-115, Ag-203, and Ag-372 samples, slice thicknesses of, respectively, 5 nm, 10 nm, and 20 nm were made. After each slice, a secondary electron SEM image at 2 kV and 50 pA was taken.

The data analysis was performed using ImageJ. For Ag-203 the obtained dataset was large enough to split it into two datasets. After some pre-processing and processing steps, which are discussed in more detail in ESI† section SI, the 3D Viewer plugin^22^ of ImageJ was used to visualize and reconstruct the 3D surface. A 3D watershed (3D ImageJ Suite)^23^ was applied to separate the individual pores and by measuring the center-to-center distances of the pores,^23^ the data for the pore distance distributions were obtained. The pore volume was calculated by using the ratio between the segmented voxel of Ag and porous space. The Skeletonize3D plugin^24^ of ImageJ was used to obtain skeletons, which were then measured and characterized through the AnalyzeSkeleton plugin. The Matlab App^25^ TauFactor^26^ was used to determine the diffusion in the *x*, *y*, and *z* directions and to determine the 3D pore volume.

To characterize the crystalline phases of the catalyst, X-ray diffraction (XRD) was performed on a Bruker D2 Phaser with a Co Kα X-ray source (1.79026 Å). The diffractograms were measured between 40° and 120° with a step size of 0.03° and a dwell time of 1 s per step and the data were normalized.

### Electrochemical performance

The electrochemical characterization and catalytic measurements were performed using a PARSTAT MC potentiostat in a three-electrode H-type cell, which was discussed in detail before in our group.^27^ A Pt disc as the counter electrode, a 3 M Ag/AgCl reference electrode, a Nafion 117 proton exchange membrane and a 0.1 M KHCO_3_ (Honeywell Fluka, 99.7%) electrolyte solution that was pretreated with Chelex® and stirred with a stirring bar at 500 rpm were used. The electrodes were placed in the cathodic compartment on top of a glassy carbon disk that was polished with a diamond suspension (MetaDi Supreme; 1 μm, 0.25 μm and 0.05 μm). Prior to the measurements, the cell was purged with CO_2_ for at least 1 h to saturate the solution. A cyclic voltammetry (CV) was run between −0.5 V and −2.0 V for at least 3 cycles to reduce any oxide layer present on the catalyst surface. Subsequently, the double-layer capacitance (DLC) was measured by performing CV in the non-faradaic regime (see Fig. S9†) between −0.3 V and −0.5 V. Different scan rates ranging from 0.02 V s^−1^ to 5 V s^−1^ were used for the CV. The current at −0.4 V *vs.* Ag/AgCl was plotted *versus* the scan rate. Only the data points from the scan rates with a symmetric CV (<0.2 V s^−1^, see Fig. S9†) were used to determine the slope of the curve (see Fig. S10†). The slope gave the electrochemical capacitance of the catalyst, which was used to calculate the surface area of the catalyst by comparing the capacitance with the specific capacitance of an Ag foil (Alfa Aesar, 99.95%) measured under the same conditions.

Thereafter, a fixed voltage (ranging from −0.7 V, −0.9 V, −1.2 V, −1.3 V and −1.4 V *vs.* RHE) was applied for 30 minutes and the current response was measured. The potentials were converted to the reversible hydrogen electrode (RHE) using the following equation:*E*_RHE_ = *E*_Ag/AgCl_ + 0.209 + 0.059 pHAfter each applied potential, electrochemical impedance spectroscopy (EIS) was measured between 1 Hz and 10^6^ Hz with a voltage amplitude of 10 mV whilst applying the same potential. Fitting the Nyquist plot with a *R*1 + *Q*2/*R*2 circuit gave both the resistance (*R*1) and the capacitance (*Q*2) of the system. For three data points, the EIS after catalysis at −1.4 V *vs.* RHE failed, based on the resistances at other potentials and from other samples, and in these cases a value of 18.5 Ω was assumed. The resistance was used to afterwards compensate the catalytic data for the uncompensated resistance (mostly caused by the electrolyte solution).

Gaseous products were online detected with a gas chromatograph (GC), using a Gas Analyzer Solution Compact Microcompact GC 4.0. This GC had three columns: a Rt-QBond (10 m × 0.32 mm, Agilent), a molecular sieve 5A (10 m × 0.53 mm, Restek) and a Carboxen 1010 (8 m × 0.32 mm, Agilent) column, connected with, respectively, a FID, a FID detector (together with a methanizer to increase the CO sensitivity) and TCD detector to measure the presence of CH_4_, C_2_H_4_ and C_2_H_6_ (first column), CO and CH_4_ (second column) and H_2_ and CO_2_ (third column). To detect liquid products, a liquid sample of 0.5 mL was taken for NMR after each applied potential. The catholyte was replenished with 0.5 mL fresh 0.1 M KHCO_3_. Liquid products were quantified with ^1^H NMR, using a 400 MHz Varian NMR with solvent suppression and 50 mM phenol and 10 mM DMSO as an internal standard solution. The current densities and product concentrations were used to determine the activity and selectivity of the catalysts.

After catalysis, Pb underpotential deposition (Pb UPD) was measured for each pore size on one sample to determine the surface area. A three-electrode setup similar to that used for Ag deposition was employed, consisting of a carbon paper (Toray TGP-H-060) anode, a 3 M Ag/AgCl reference electrode and a glassy carbon disc with the template-based Ag electrode as the cathode. A solution of 0.01 M Pb(NO_3_)_2_ (Puratronic®, 99.999%) in 0.01 M HNO_3_ (VWR Chemicals, 65%) was used as the electrolyte, and CVs were measured between 0 V and −0.5 V *vs.* Ag/AgCl with a scan rate of 1 mV s^−1^ for 3 cycles. Only the results from the second and third cycles were used, as the first cycle also included currents caused by the formation of the electrical double-layer and reduction of Ag_2_O. The surface area was obtained by integrating the underpotential deposition peak around −0.3 V *vs.* Ag/AgCl in the cyclic voltammogram and converting this into an area using the scan rate and a correction factor, derived from Pb UPD on flat Ag, of 1.67 × 10^−3^ cm^2^ μC^−1^.^28^

## Results and discussion

### Poly(methyl methacrylate) sphere templates

**Fig. 2:**
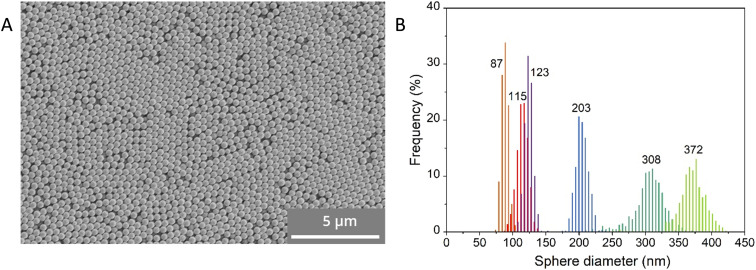
A) SEM image of sample PMMA-372 showing PMMA spheres with an average diameter of 372 nm; B) diameter distributions of the PMMA spheres prepared under slightly changing reaction conditions. The numbers indicate the average diameter in nm.

Poly(methyl methacrylate) (PMMA) sphere templates with different sizes were synthesized under slightly different conditions (Table 1) and named PMMA-87, PMMA-115, PMMA-123, PMMA-203, PMMA-308 and PMMA-372, with the numbers symbolizing the average diameter of the PMMA spheres. Using scanning electron microscopy (SEM), the morphology and sphere diameter of the PMMA samples were determined. In Fig. 2A, a SEM image of sample PMMA-372 is given as a representative example. The image shows stacked spheres forming domains with ordered face-centered cubic (FCC) structures. The other PMMA samples showed a similar structure (Fig. S1†). The resulting sphere diameter distributions are given in Fig. 2B. As this figure shows, the six samples yielded PMMA spheres with average diameters of 87 nm, 115 nm, 123 nm, 203 nm, 308 nm, and 372 nm. All the PMMA samples had a polydispersity index (PDI) below 0.04 (see Table 1) indicating that the samples were sufficiently monodisperse to form opal structures.^21^

### Porous Ag electrodes

**Fig. 3:**
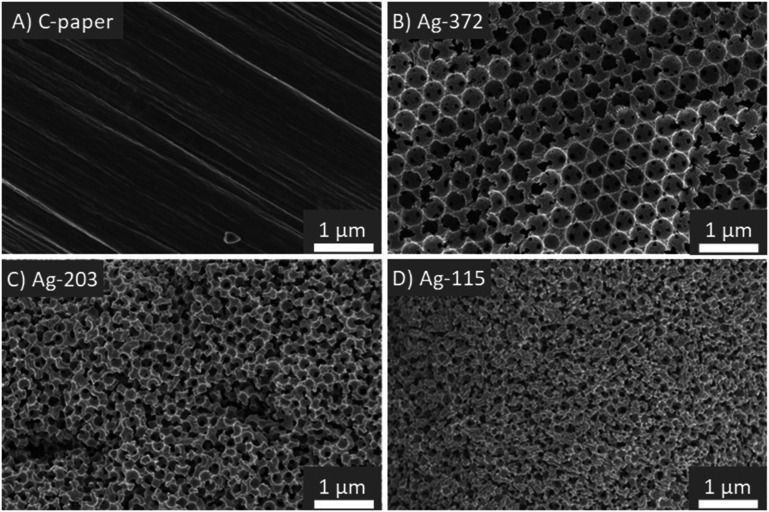
A) Bare carbon paper used as the conductive substrate for the electrodes; porous Ag electrodes made by electrodeposition of Ag on a PMMA sphere template with a sphere diameter of B) 372 nm, C) 203 nm and D) 115 nm on carbon paper as the substrate.

Porous Ag electrodes were obtained *via* Ag deposition and subsequent removal of the PMMA template. These were named Ag-87, Ag-115, Ag-123, Ag-203, Ag-308 and Ag-372, with the number corresponding to the sphere diameter of the PMMA template used (the real pore diameter is ∼10% lower, see Fig. S2†). Fig. 3 shows representative SEM images of the bare carbon paper (A) and the porous Ag samples Ag-372 (B), Ag-203 (C) and Ag-115 (D). The SEM images show that when the diameter of the template sphere decreases, the pore diameter decreases as well. Apart from that, the overview images (Fig. S3†) show good coverage and attachment of the carbon fibres with silver for all Ag samples, except for Ag-87. Despite multiple attempts, it was difficult to obtain good coverage for the Ag-87 sample. It seems that the voids between the spheres of this finest template are too small to achieve appropriate Ag deposition. Hence, our synthesis route combining Ag electrodeposition with templating has a lower limit in the pore size for which it can be used. In XRD (Fig. S4†), all the peaks were attributed to either crystalline Ag (45°, 52°, 77°, 93° and 99°) or the carbon paper substrate (64°).

Remarkably, it is possible to make template-based porous catalysts on non-flat materials, such as carbon paper consisting of a fibre-like structure. This has not been described in the literature before, but can be relevant for research and industrial applications where non-flat electrode materials such as carbon paper or carbon cloth for GDEs are used.

### Influence of pore size on electrochemical CO_2_ reduction

The electrodes were tested for CO_2_ reduction at five different potentials: −0.7, −0.9, −1.2, −1.3 and −1.4 V *vs.* RHE, which were *iR* compensated during the data analysis (with an error of <0.1 V). For the samples Ag-203, Ag-308 and Ag-372 the measurements were performed in *duplo*. The Ag-115 and Ag-123 samples had similar pore sizes and overlapping pore size distribution (Fig. 2B). These samples were made to prove the reproducibility of the whole synthesis route. Therefore, in the catalytic performance these two samples were considered a duplicate. Due to the poor Ag coverage as described above, the catalytic performance of the Ag-87 catalysts was not measured. All electrodes gave stable current responses with minor fluctuations (<10%), which might be ascribed to the bubble formation (Fig. S5†). All five catalysts were active in the CO_2_ reduction reaction, producing CO. Apart from that, H_2_ was formed as a side product. Other gas products (CH_4_ and C_2_H_4_) were not detected in significant amounts (<0.2%). For the Ag-203 and Ag-372 samples, NMR was measured, showing the formation of formate. This liquid product was only formed in low amounts (<7.5% FE, see Table S1 and Fig. S6†). Hence, it will not be discussed further. The faradaic efficiencies of CO and H_2_ are given in Fig. S7.†

**Fig. 4:**
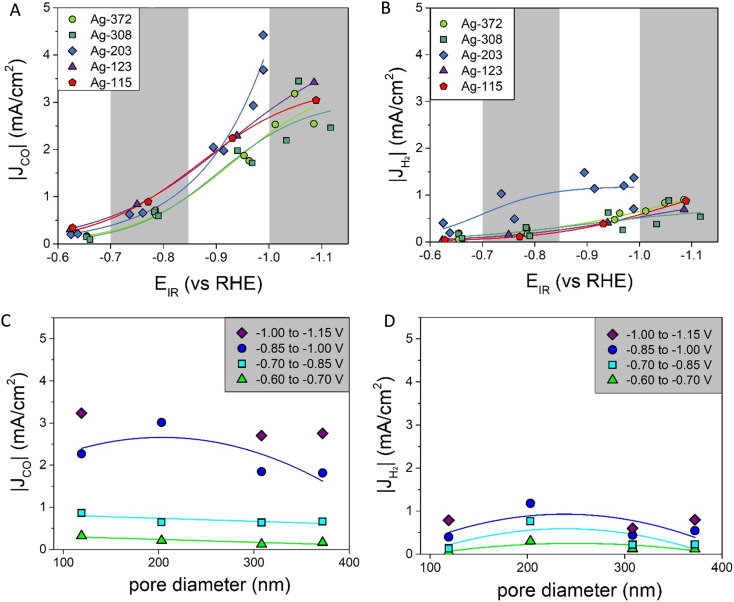
A) Partial current densities of CO at different potentials fitted with a sigmoidal function (for more details see ESI† part G); B) partial current densities of H_2_ at different potentials fitted with a sigmoidal function (for more details see ESI† part G); C) partial current densities of CO for different pore diameters fitted with a linear (−0.60 to −0.70 V and −0.70 to −0.85 V) or parabolic function (−0.85 V to −1.00 V) as a line to guide the eye; D) partial current densities of H_2_ for different pore diameters fitted with a parabolic function as a line to guide the eye.

In Fig. 4A and B the partial current densities of, respectively, CO and H_2_*vs.* the potential are shown. All porous Ag samples produce significantly more CO than H_2_, up to 4 times more. The Ag-203 catalyst is the most active, producing both more CO and H_2_, in particular at more cathodic potentials below −0.9 V *vs.* RHE. Apart from the Ag-203 catalyst, the |*J*_CO_| for all catalysts saturates at higher current densities, indicating that mass transfer limitations start to play a role.

To better visualize the dependence on the pore size, Fig. 4C and D show the partial current densities *versus* the pore diameter for CO and H_2_, respectively. In these graphs multiple datapoints for each pore size are averaged. For CO, there is a dependence of |*J*_CO_| on the pore size. For the moderate potentials (between −0.60 V and −0.85 V *vs.* RHE) a decrease in |*J*_CO_| was found when increasing the pore diameter. At more cathodic potentials (below −0.85 V *vs.* RHE), the data suggest an optimum, with the highest current density found for pore sizes around 200 nm. Also, for H_2_, there seems to be a slight optimum around a pore size of 200 nm for all the potentials. However, it is important to note that H_2_ production is lower, which means the absolute differences between the various pore sizes are also less pronounced. These trends in the partial current density depending on the pore diameter can be compared to the faradaic efficiencies *versus* the pore diameter, which are given in Fig. S7i and j.† Interestingly, the faradaic efficiency of CO is lower for the catalysts with a pore size of ∼200 nm.

**Fig. 5:**
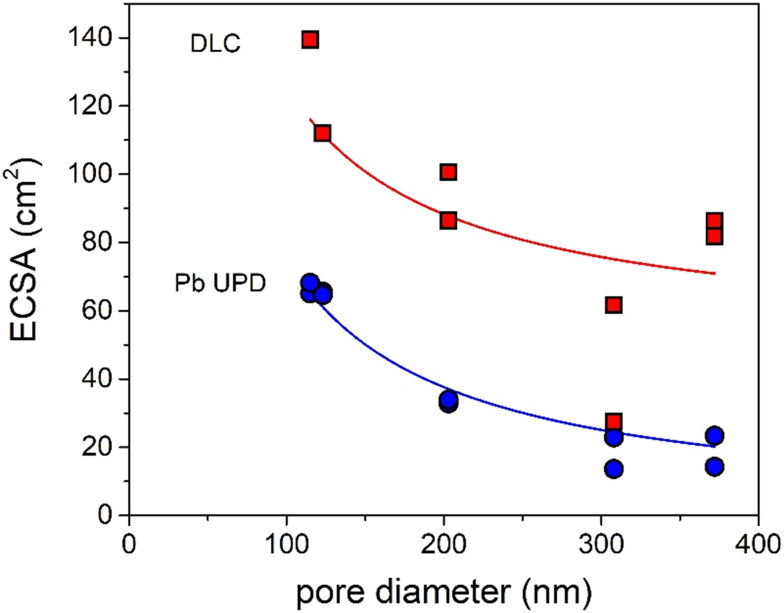
Surface area of the Ag samples determined *via* Pb UPD (blue) and DLC (red) plotted *versus* their pore diameters fitted with a *y* = *a*/*x* + *b* function.

How can these effects be explained? First of all, the graphs in Fig. 4 show the partial current density per geometric surface area. Increasing the pore diameter leads to a decrease in the electrochemical surface area (ECSA). Hence, for fundamental understanding, it is more meaningful to normalize the partial current density to the ECSA. To determine the ECSA, both Pb underpotential deposition (UPD) and double-layer capacitance (DLC) were used. Pb UPD was performed on the porous Ag samples after catalysis. In Fig. S8,† the Pb underpotential deposition curves are shown. The ECSA was obtained by the integration of the peak areas and conversion into an area (for details see Experimental). In Fig. 5, the ECSAs for the five different samples are plotted against the pore diameter and they are given in Table S3.† The trend in the surface area scales inversely with the diameter of the pores, as is expected. These values were compared with the capacitance values found with DLC, which are plotted in Fig. 5 and given in Table S3.† These surface areas are much higher. This can be explained by the fact that the bare C-paper has a much higher capacitance than Ag (see Fig. S10†). Ag was only deposited from the front side, so towards the back, some C-fibers can be found that are not covered with porous Ag (see Fig. S3†). Hence, a higher off-set value had to be used in the fitting to correct for this constant, additional capacitance. To make sure to only account for the Ag surface area, the Pb UPD results were used to normalize the ECSA.

**Fig. 6:**
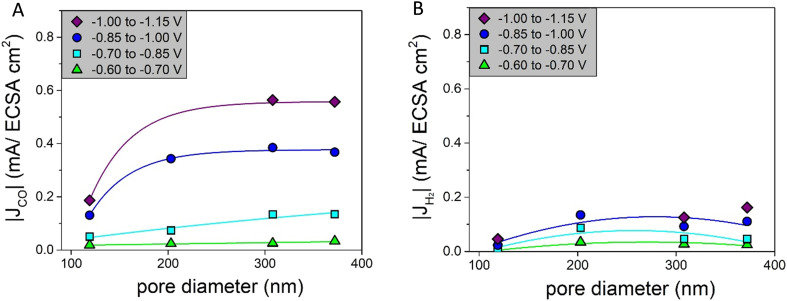
A) Partial current densities of CO normalized by the ECSA for different pore diameters fitted with an asymptotic function as a line to guide the eye; B) partial current densities of H_2_ normalized by the ECSA for different pore diameters fitted with a parabolic function as a line to guide the eye.

The intrinsic activities, defined as the ECSA-corrected partial current density graphs, are shown in Fig. 6A for CO and Fig. 6B for H_2_. Fig. 6A shows that for |*J*_CO_| there is a clear pore size dependence: up to a pore size of ∼300 nm, there is an increase in the partial current density upon increasing the pore diameter, and for the pore sizes above ∼300 nm, the partial current density does not change further. The H_2_ production in Fig. 6B first increases strongly up to a pore size of ∼200 nm and decreases slightly afterwards. So, both the intrinsic CO and H_2_ production depend on the pore size.

Interestingly, these results differ from predictions in the literature. Suter *et al.* predicted that a smaller pore diameter changing from 400 nm to 100 nm would lead to higher concentrations of OH^−^ and CO_3_^2−^ and therefore increase |*J*_CO_|.^8^ However, we cover both low and intermediate overpotentials, hence capturing pore size effects over the full relevant potential range. It is known that the formation of CO and H_2_ on a Ag catalyst is potential dependent, with high H_2_ production at low overpotentials and high CO production at intermediate overpotentials.^29^ At intermediate potentials pore size effects on the CO production will be less pronounced, as the CO production is already dominant. This can explain part of the differences.

Furthermore, the tortuosity and length of the pore network of the catalysts are important parameters to consider when understanding these results. Although the pore sizes in our system are well defined, the porosity in the samples studied is not always perfectly homogeneous, in particular not for the smaller pore sizes. This can influence the tortuosity and the pore network length of the catalysts. A catalyst that is more tortuous has a lower effective diffusion coefficient, which increases CO formation while suppressing H_2_ formation, because of a higher local pH.^7^ Related to this, additional ohmic resistances can be present for longer pores and influence the system when current is present.^7^ Hence, in the next section, we will discuss the tortuosity and pore length of our catalysts in more detail.

### Porosity analysis

**Fig. 7:**
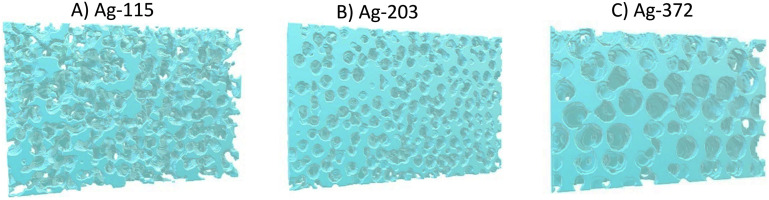
3D reconstruction based on the FIB-SEM slice-and-view results from the samples A) Ag-115; B) Ag-203; and C) Ag-372.

To obtain more detailed information about the porosity of the catalysts and in particular the tortuosity and the pore network length, a detailed structural analysis was performed using focused-ion beam scanning electron microscopy (FIB-SEM). FIB-SEM has been used before to characterise (porous) CO_2_ reduction catalysts,^30–32^ but to our knowledge, it has not been used before to investigate ordered, porous CO_2_ reduction catalysts. Pristine Ag-115, Ag-203, and Ag-372 were analysed. The sizes of the sample porous electrodes analysed are given in Table 2. The full electrode has a geometric surface area of 3.8 cm^2^ and a thickness of 190 μm. Fig. 7 shows representations of the 3D reconstructions that were made for the samples Ag-115 (A), Ag-203 (B), and Ag-372 (C) and the full 3D reconstructions can be found in ESI† Movies S1–S4. For the Ag-115 catalyst, pores matching the size and shape of the PMMA sphere template were observed, along with larger voids. These larger voids are likely the result of imperfect Ag deposition within the voids of the PMMA sphere template. This observation supports the previous conclusion that for smaller spheres templated electrodeposition is less successful as a synthesis method. For the Ag-203 catalyst, the observed pores predominantly match the size and shape of the PMMA spheres used. For the Ag-372 catalyst, pores consistently match the size and shape of the PMMA spheres. To express these observations more quantitatively, we can have a look at the pore distance distribution graphs in Fig. S11.† For a perfect monodisperse FCC structure based on monodisperse spheres, we expect to only see peaks at multiples of *d*, the pore diameter. For the Ag-115 catalyst, these peaks are not found. Therefore, there is no quantifiable order. For the Ag-203 catalyst, a peak around 200 nm, the diameter, is observed, but no peaks are observed for 2*d*, 3*d*, *etc.* So the order is considered to be up to the nearest neighbors. For the Ag-372 catalyst, the order extends beyond the next–next nearest neighbours, as evidenced by the peaks around 370 nm, 740 nm and 1110 nm—corresponding to *d*, 2*d*, and 3*d*. The calculated pore volumes (Table 2) are in line with these results. Based on a packing factor of 0.74 for a FCC structure and the density of Ag, a pore volume of 29.9 cm^3^ g^−1^ Ag is expected. For the Ag-372 catalyst, the volume is similar to this. For the Ag-203 catalyst, it is lower, which is in line with the absence of order larger than one nearest neighbour mentioned above. For the Ag-115 catalyst, it is close to the pore volume FCC structure, despite the absence of order. This can be explained by the observation that larger voids are present, leading to a higher pore volume.

**Table 2:** Properties obtained from the 3D model based on the FIB-SEM data for the Ag-115, Ag-203 and Ag-372 catalysts

Electrode	Size dataset (*l* × *w* × *d* in μm)	Pore volume (cm^3^ g^−1^ Ag)	Diffusional tortuosity	Diffusional tortuosity	Diffusional tortuosity	Theoretical pore network length (μm μm^−3^)	Reconstructed pore network length (μm μm^−3^)
			*τ*_*x*_	*τ*_*y*_	*τ*_*z*_		
Ag-115	3.00 × 2.00 × 0.58	24.8	1.45	1.58	1.35	642	117
Ag-203	3.00 × 2.00 × (0.77 and 0.85)	12.6	2.41	3.30	1.80	206	117
Ag-372	3.00 × 2.00 × 1.31	28.1	1.49	1.89	1.58	61	65

Using these 3D reconstructions the diffusional tortuosity through the catalysts was calculated. In Fig. S12 and Movies S5–S8,† the calculated diffusion through the 3D reconstructions is depicted. The blue colour indicates a low flux density, whereas the orange/yellow colour indicates a high flux density. The movies show that for all three samples, the bottleneck of the diffusion lies at the pore window connecting two adjacent pores. In Table 2, the diffusional tortuosity in three different directions (*x*, *y*, and *z*) is given. The tortuosity in the *x* direction is particularly important, as this represents the primary pathway from the bulk electrolyte into the porous catalyst. The Ag-203 catalyst has the highest tortuosity, namely, 2.41 compared to 1.45 and 1.49 for, respectively. Ag-115 and Ag-372. As a higher tortuosity is known to increase CO production due to a local higher pH,^7^ this can explain the higher |*J*_CO_| in Fig. 6A at ∼200 nm compared to smaller pores. However, it does not explain why the CO production keeps increasing between ∼200 nm and ∼300 nm. Also, the increase in CO production is usually accompanied by a decrease in H_2_ production,^7,8,16^ which is not observed in Fig. 6B. This indicates that tortuosity is not the sole factor influencing the catalytic performance of the system.

The pore network length is defined as the total length of all pore-to-pore connections normalized to the volume. For a perfect FCC structure, the pore network length can easily be calculated: each pore has 12 nearest neighbours at a distance *d*, the diameter. Counting each connection once in a unit cell with 4 pores and a volume of 
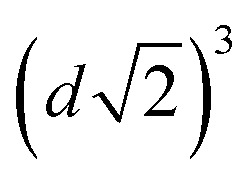
, this gives a total pore network length of 
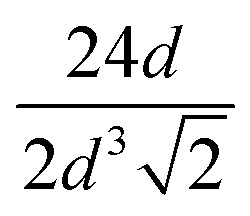
 in m m^−3^. For the Ag-115, Ag-203 and Ag-372 catalysts, the theoretical pore network lengths are given in Table 2 and are, respectively, 632 μm μm^−3^, 206 μm μm^−3^ and 62 μm μm^−3^. The pore network lengths that were reconstructed *via* skeletonization of the porous structure (see Fig. S13 and Movies S9–S12†) are also given in Table 2. These are 117 μm μm^−3^, 117 μm μm^−3^ and 65 μm μm^−3^ for, respectively, the Ag-115, Ag-203 and Ag-372 catalysts.

Two aspects of these outcomes are very remarkable. First of all, the smaller the pore diameter, the larger the difference between the theoretical and the reconstructed pore network length. For the Ag-115 catalyst, the reconstructed pore network length is 5.5 times shorter than the predicted pore network length. This is an additional measure to quantify the level of order in the catalysts. Secondly, it is interesting to see that, when comparing the catalysts, the larger the pore diameter, the shorter the pore network length. This is a difference of more than a factor of 2. It is known that longer pore networks lead to additional potential drops during electrochemical measurements, as not only the resistance of the bulk electrolyte (*R*_sol_) but also the diffusion resistance in the electrolyte inside the pores (*R*_pore_) has to be taken into account.^7^ However, the *iR* correction only includes *R*_sol_. Therefore, it is likely that for Ag-203 and Ag-115, a higher additional potential drop is present than in the Ag-372 catalyst. This also implies that for these two catalysts effectively a lower potential is applied inside the pores, especially when larger currents are drawn. This explains why catalysts with smaller pore diameters have a lower intrinsic activity Fig. 6A. As the Ag-115 and Ag-203 catalysts have equal pore network lengths, but do not have the same CO production in Fig. 6A, the pore network length is not the only factor influencing the intrinsic activity. So to conclude, the FIB-SEM data and analysis help to understand that the catalytic performance is not only influenced by the pore size, but also by the tortuosity and additional Ohmic resistances, especially when it comes to the intrinsic activity. The CO production found in Fig. 4C is a combination of an increased number of active sites for smaller pore sizes caused by a larger surface area and a decreased intrinsic activity due to the tortuosity and additional potential drops, leading to a maximum in activity at a pore size of ∼200 nm.

### Catalyst stability

**Fig. 8:**
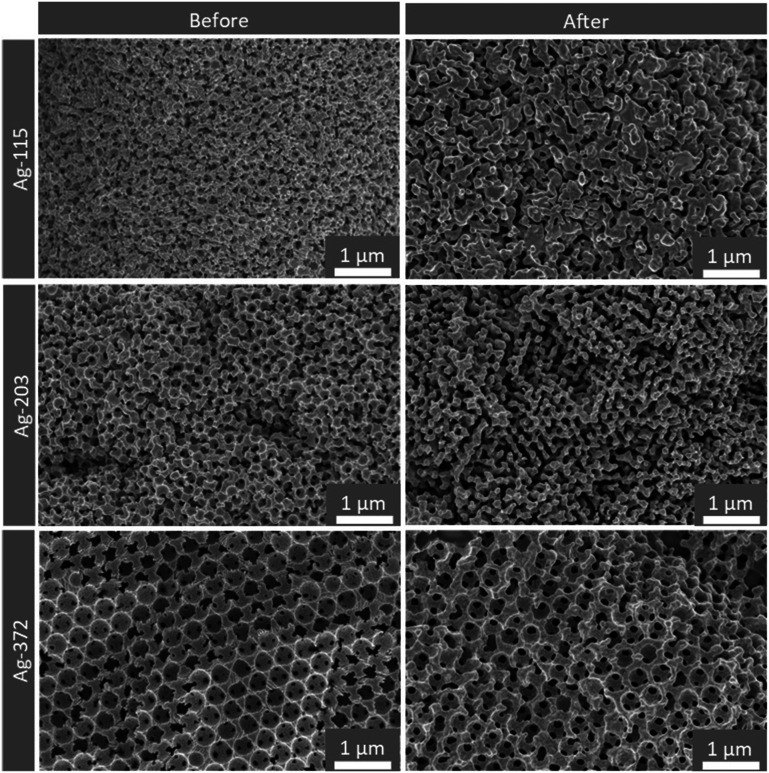
SEM images of Ag-115, Ag-203 and Ag-372 nm before (left) and after at least one cycle of catalysis (right).

Having discussed the catalysts' activity and selectivity, it is also important to assess the stability of the catalysts. XRD measurements performed after 2 h of catalysis are given in Fig. S4.† The diffractograms do not show any significant change before and after catalysis. When considering the SEM images after catalysis (Fig. 8 and S14†), it can be seen that the Ag electrodes preserve their porous structure. However, the edges of the pores became smoother, and the pores are less defined, which is caused by applying (more) negative potentials during the CV and the catalytic testing.^17^ From a thermodynamic perspective, these structural changes are expected, favouring spherical shapes over sharp edges and atoms with low coordination numbers. This effect appears stronger for smaller pores. However, for all three samples, the changes are on the 10 nm scale. Only, for the smaller pores, the relative change compared to the pore size is larger. When performing long term measurements (10 h) in a flow cell (Fig. S15†) both the faradaic efficiencies and the current density do not change over time. Although this is a first indication of stability, much longer tests are needed to investigate whether these catalysts are suited for industrial application. Even without industrial application, these porous model systems have proven to be highly interesting to obtain fundamental insights on the effect of pore size for CO_2_ reduction.

## Conclusions

Porous Ag electrodes with pore diameters between 115 nm and 372 nm were obtained. All the porous samples produce more CO than H_2_, up to 4 times more CO at the most cathodic potential around −1.05 V *vs.* RHE. We found that the intrinsic CO production depends on the pore size, as it increases when changing the pore diameters from ∼100 nm to ∼300 nm. At larger pore diameters, the pore size does not affect the intrinsic CO production anymore. For the first time, FIB-SEM was used to reconstruct the templated porous electrodes for CO_2_ reduction and to quantify the tortuosity and pore network length. The catalyst with a pore diameter of ∼200 nm had the highest tortuosity of 2.41 and this leads to an increased CO production. At the same time, the catalysts with a pore diameter below ∼200 nm have pore network lengths that are twice as long as the pore network lengths of the catalysts with an ∼400 nm pore diameter. This increase leads to an additional potential drop inside the pores when the current is drawn, which lowers the applied potential. So, we show that being able to analyse the porous structure in detail helps to disentangle the effect of pore size, tortuosity and pore network length. These results are important for designing porous catalysts with high activity and selectivity.

## Conflicts of interest

There are no conflicts to declare.

## Supplementary Material

RE-010-D5RE00068H-s001

## Data Availability

The data from this article are available at: https://doi.org/10.34894/Z40EMS (Vink-van Ittersum, Maaike; de Jongh, Petra, 2025, “Quantification of the porosity in template-based ordered porous Ag electrodes and its effect on electrochemical CO_2_ reduction”, https://doi.org/10.34894/Z40EMS, DataverseNL).
