# Supplementary material for: Quantification of the porosity in template-based ordered porous Ag electrodes and its effect on electrochemical CO2 reduction
Source: React Chem Eng. 2025 Jun 25;10(10):2300–10. doi: 10.1039/d5re00068h (PMC12188718; doi:10.1039/d5re00068h)
Supplement: RE-010-D5RE00068H-s001 [file RE-010-D5RE00068H-s001.pdf]

Supplementary Information (SI) for Reaction Chemistry & Engineering.
This journal is © The Royal Society of Chemistry 2025

Quantification of the porosity in template-based ordered porous Ag electrodes and its effect on electrochemical CO_2 reduction

Maaike E.T. Vink - van Ittersum[1], Erik Betz-Güttner[1], Eric Hellebrand[2], Claudia J. Keijzer[1], Matt L. J. Peerlings[1], Peter Ngene[1], Petra E. de Jongh[1]

[1)]*Materials Chemistry & Catalysis, Debye Institute for Nanomaterials Science, Utrecht University, Universiteitsweg 99, 3584CG Utrecht, The Netherlands*
[2)]*Department of Earth Sciences, Utrecht University, The Netherlands*

Index

A. PMMA spheres

B. Porous Ag

C. XRD graphs

D. Currents catalysis

E.NMR

F. Faradaic Efficiency

G. Sigmoidal fit catalytic data

H. Surface area

I. FIB-SEM

J. After catalysis

K. Long term catalytic performance

References

The movies S1-S12 can be found at:

Vink-van Ittersum, Maaike; de Jongh, Petra, 2025, "Quantification of the porosity in template-based ordered porous Ag electrodes and its effect on electrochemical CO2 reduction", https://doi.org/10.34894/Z40EMS, DataverseNL

A. PMMA spheres

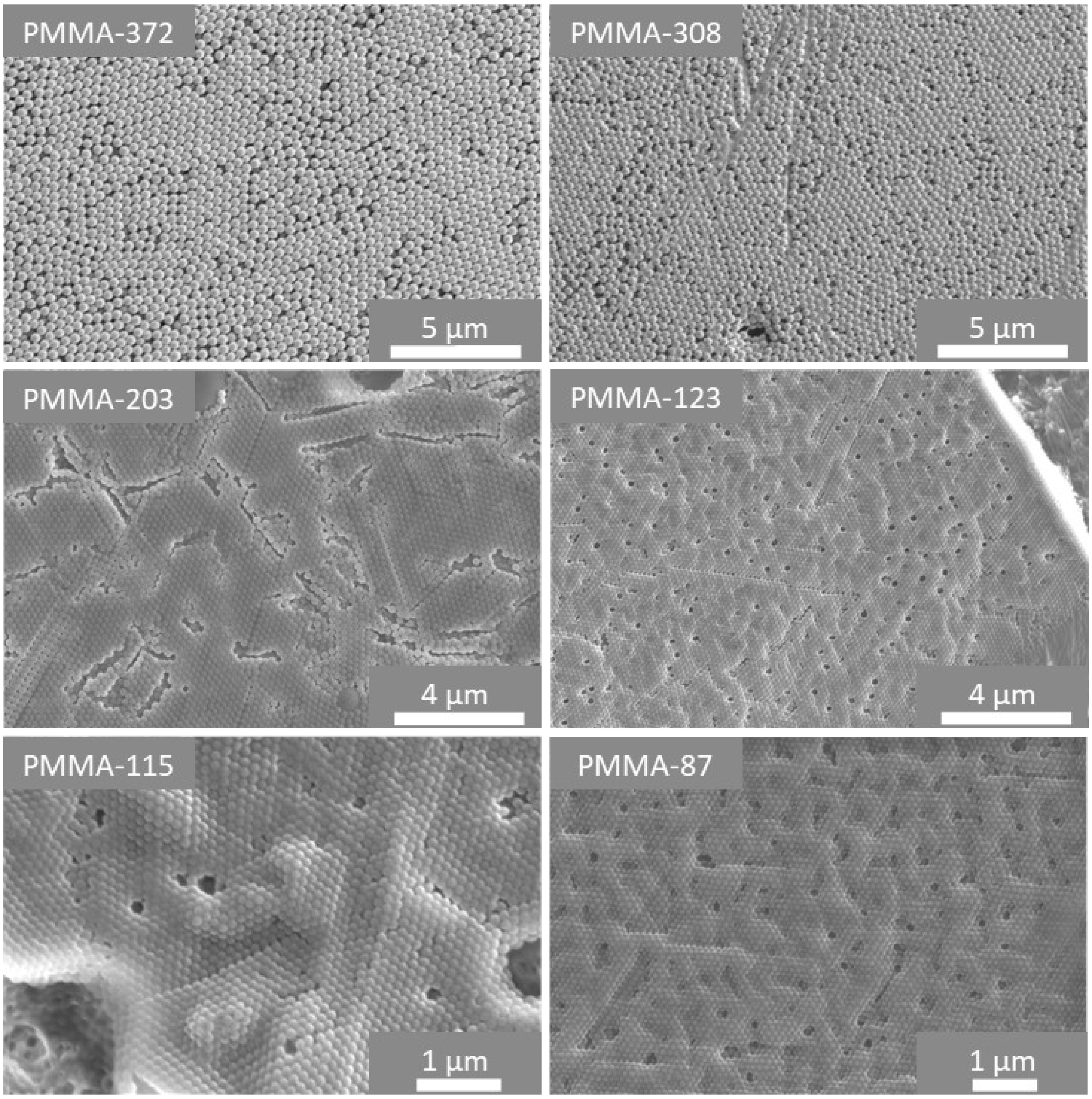

Figure S1: SEM images of the PMMA spheres in the samples PMMA-372, PMMA-308, PMMA-203, PMMA-123, PMMA-115 and PMMA-87

B. Porous Ag

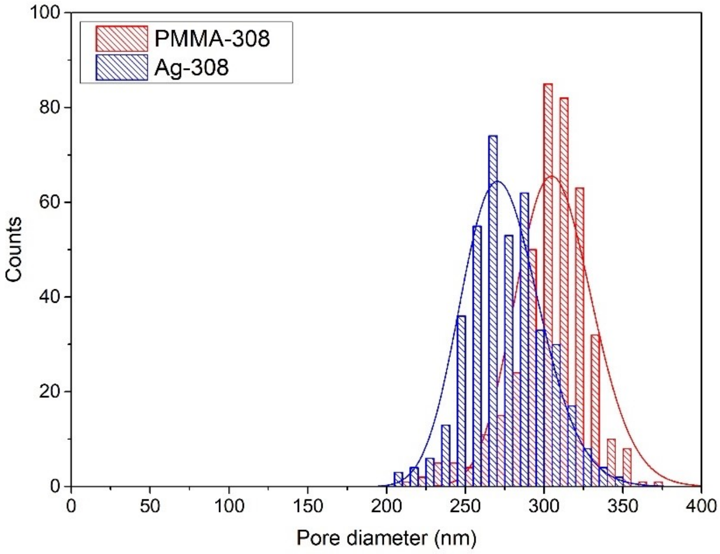

Figure S2: sphere diameter distribution curve of PMMA-308 and pore size distribution curve of a Ag-308 electrode. The pore size for the porous Ag-208 electrode is ~10% smaller than the PMMA sphere size. This is caused by shrinkage of the structure during drying.

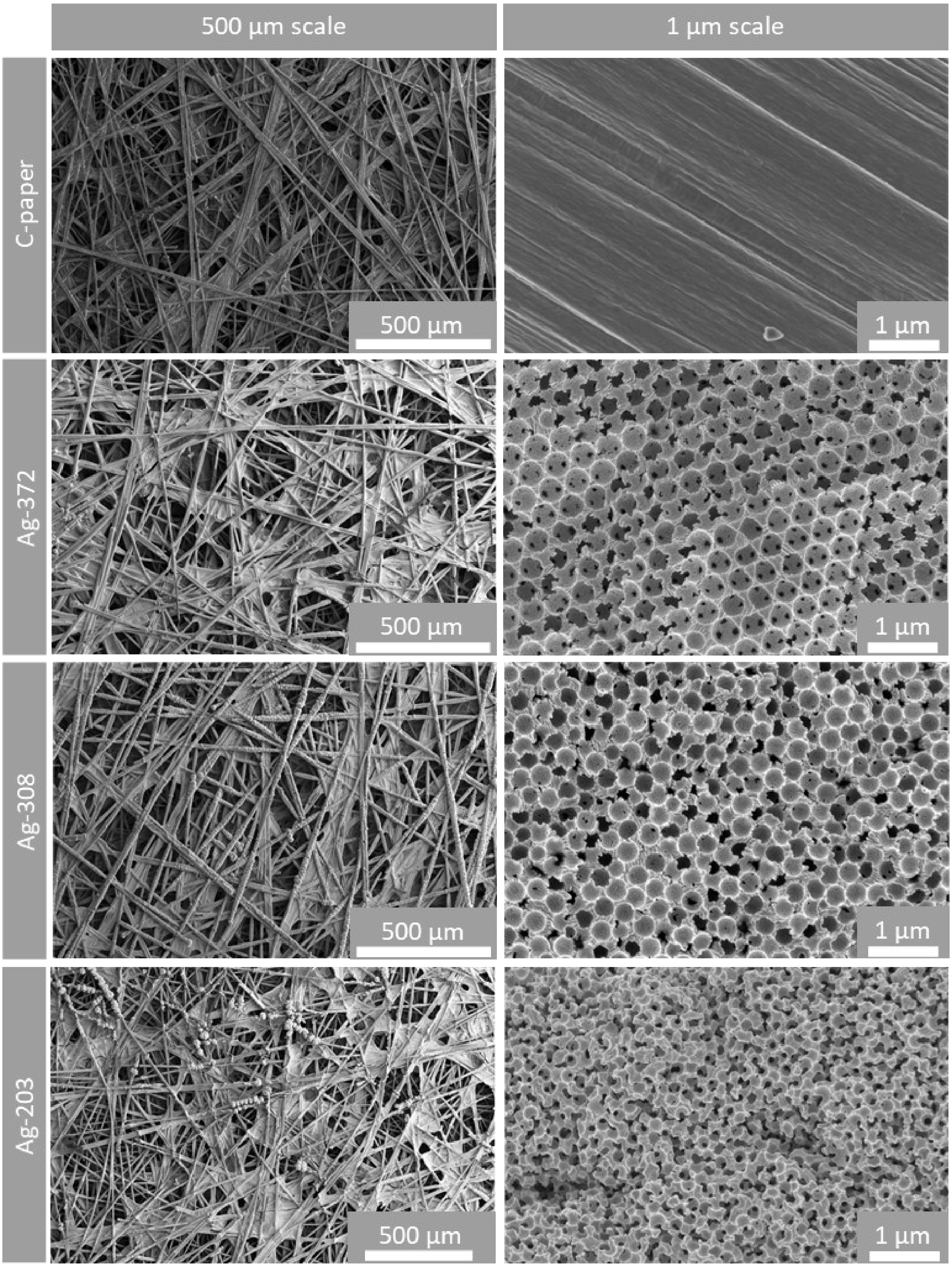
500 μm scale
1 μm scale
C-paper
Ag-372
Ag-308
Ag-203
500 μm
1 μm
500 μm
1 μm
500 μm
1 μm
500 μm
1 μm

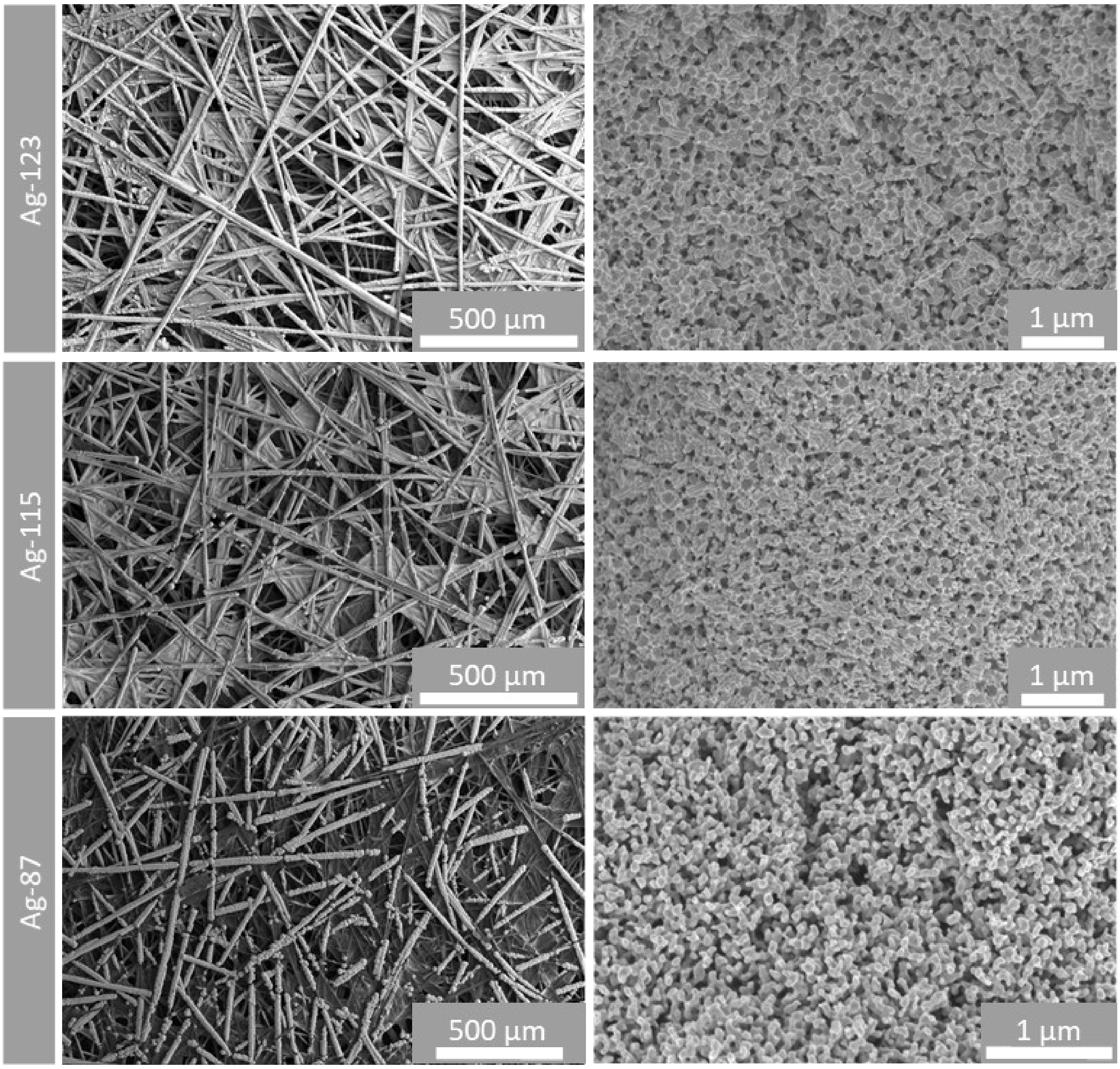

Figure S3: SEM images of bare carbon paper and the Ag-372, Ag-308, Ag-203, Ag-123, Ag-115 and Ag-87 nm samples at both 500 µm and 1 µm scale.

C. XRD graphs

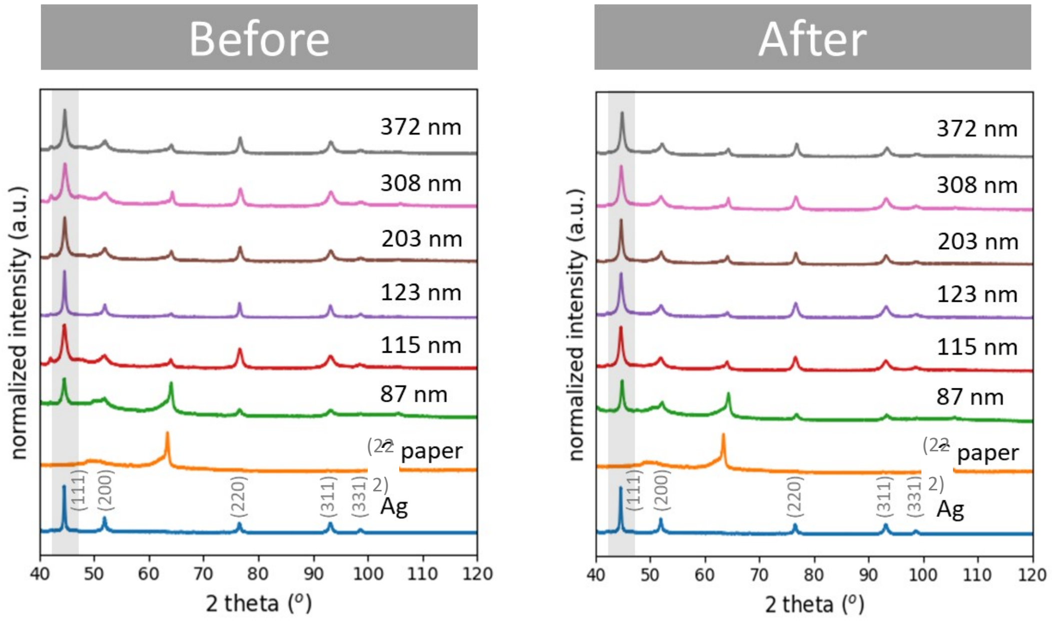

Figure S4: X-ray diffractograms of the Ag samples, carbon paper and pure Ag before and after catalysis

D. Currents catalysis

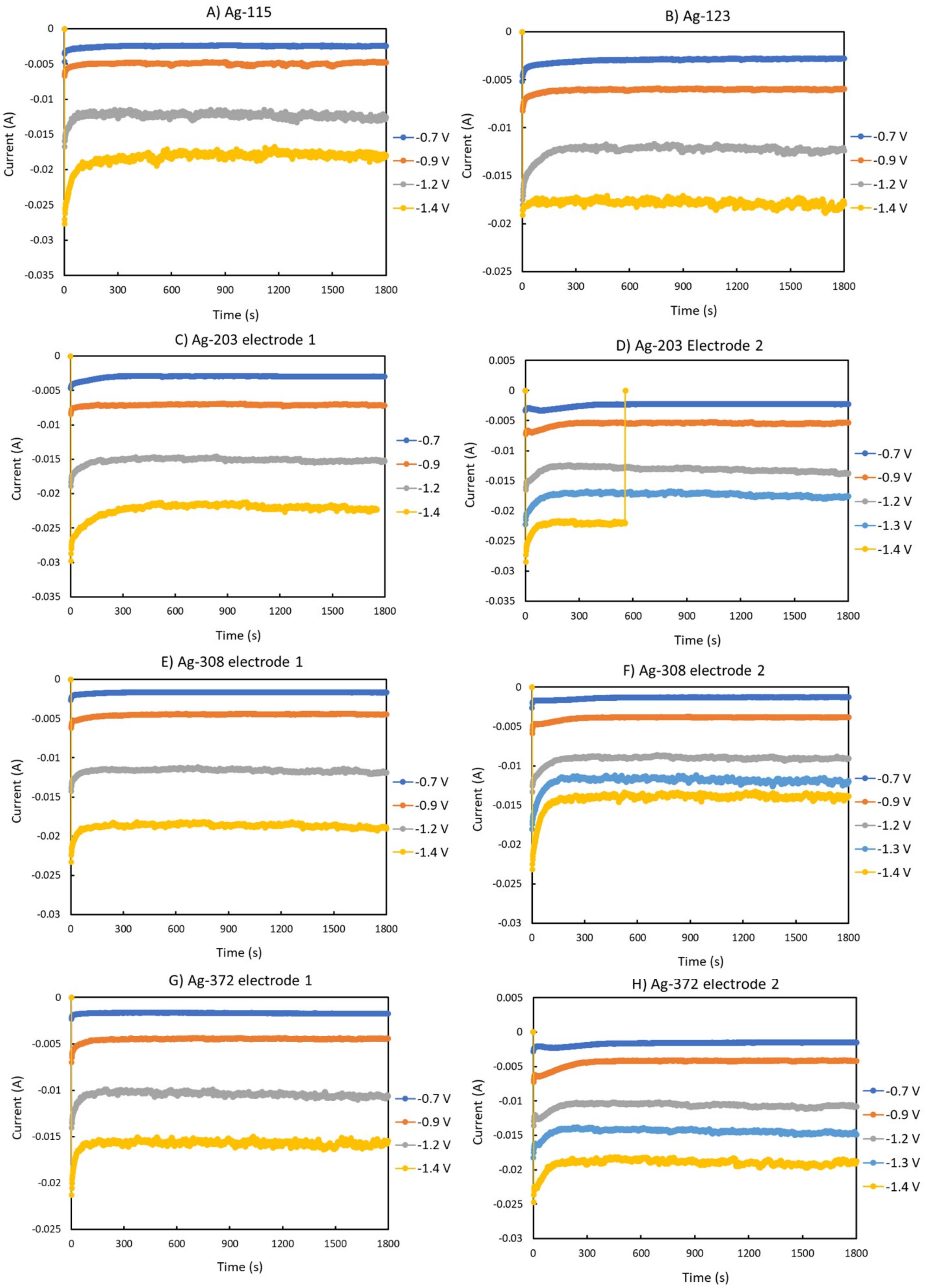

Figure S5: Current responses for the a) Ag-115, b) Ag-123, c,d) Ag-203, e,f) Ag-308 and g,h) Ag-372 catalysts when applying different potentials in catalysis. The fluctuations in the current are ascribed to bubble formation.

E. NMR

Table S1: Faradaic efficiencies of the liquid products formate and methanol formed by the Ag-203 and Ag-372 catalysts at the five different catalysis potentials.

Potential (V vs RHE)	Ag-203 FE formate (%)	Ag-372 FE formate (%)
-0.7	0	0
-0.9	0	0
-1.2	0	7.2
-1.3	0	4.4
-1.4	6.1	0

A

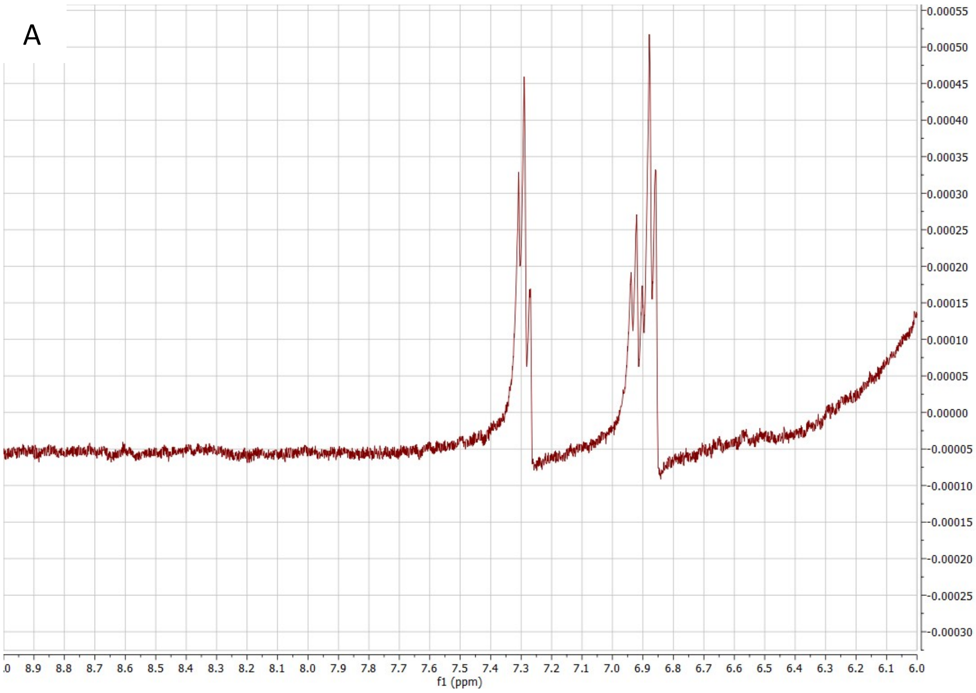

B)

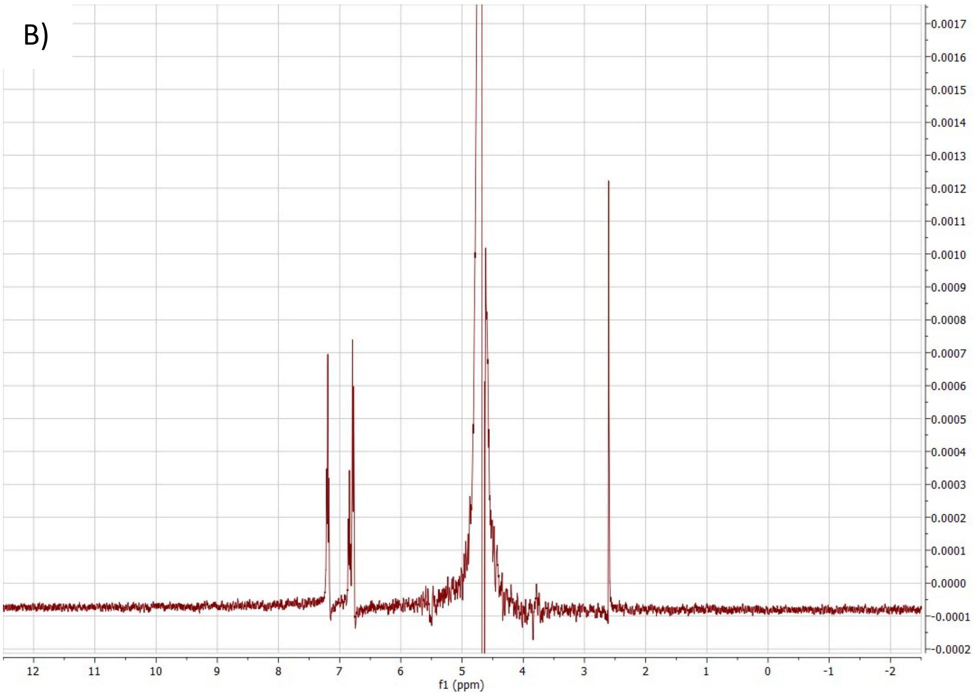

C)

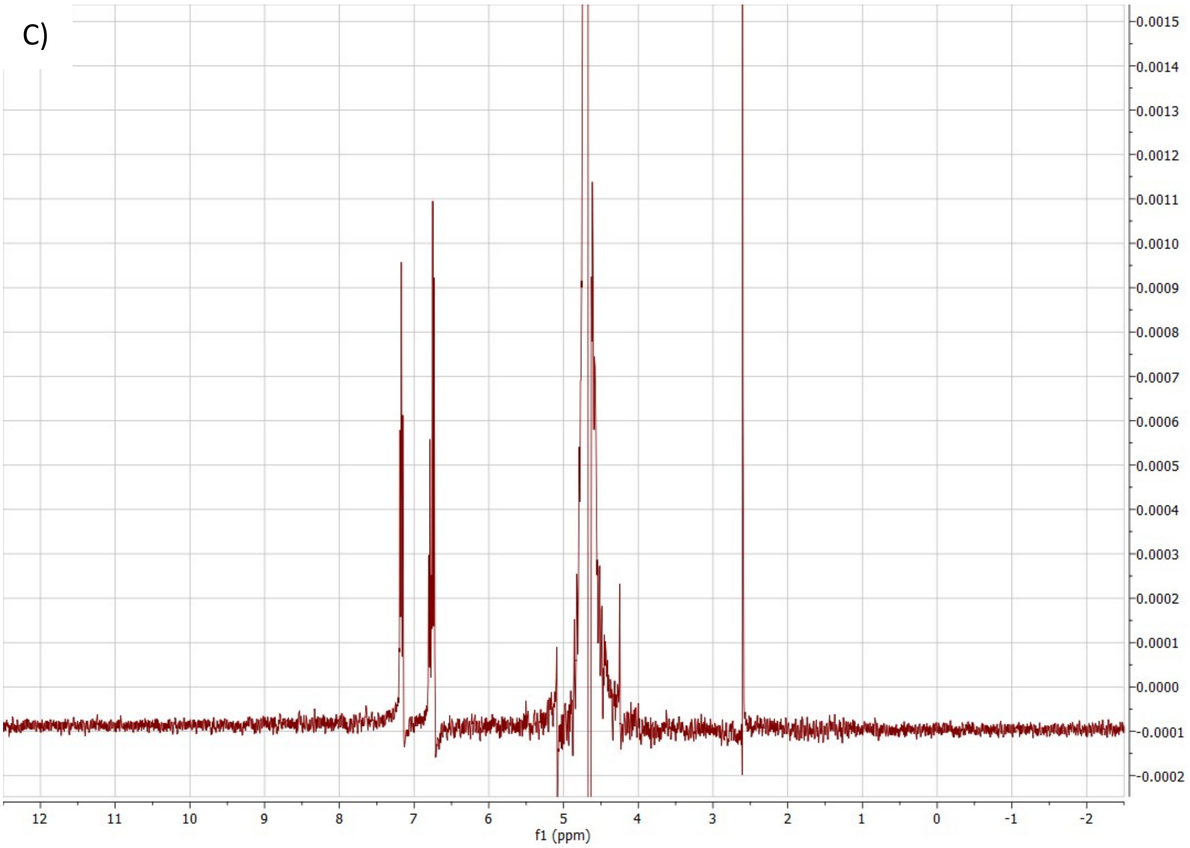

D)

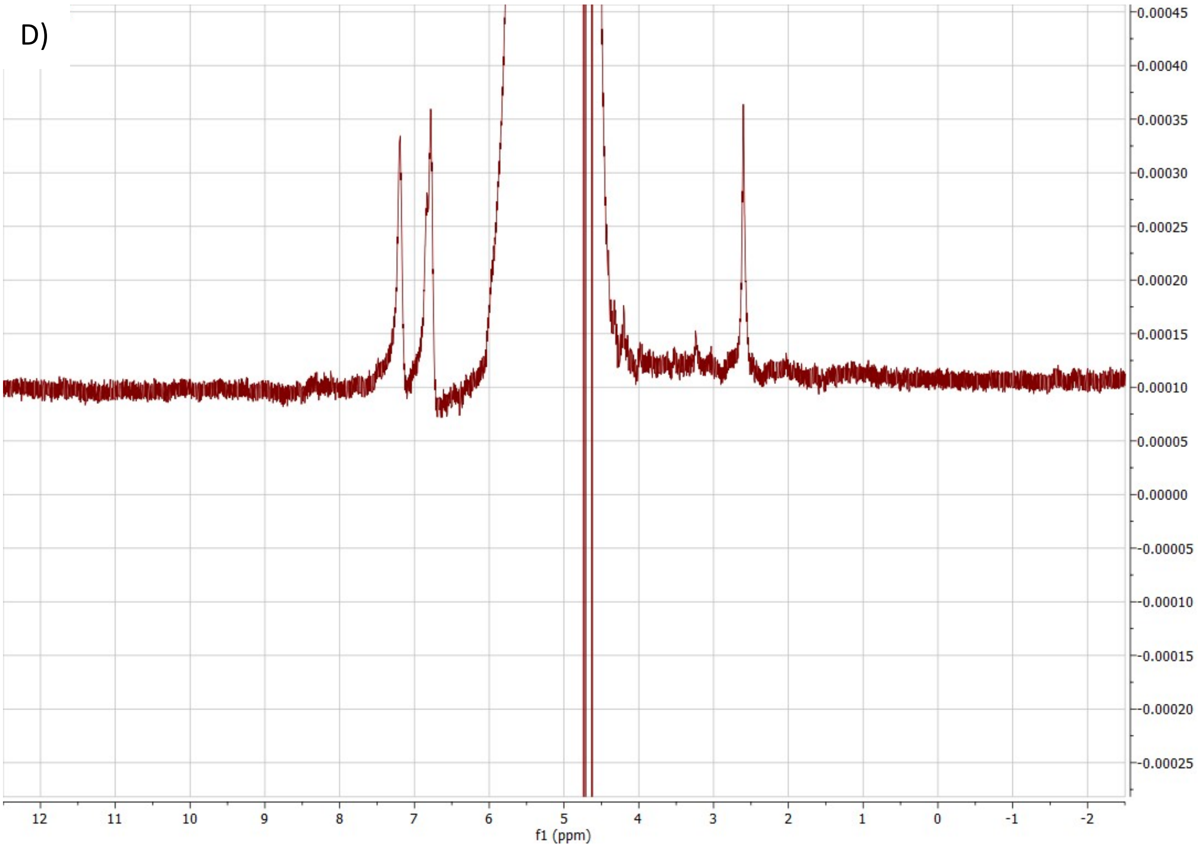

E)

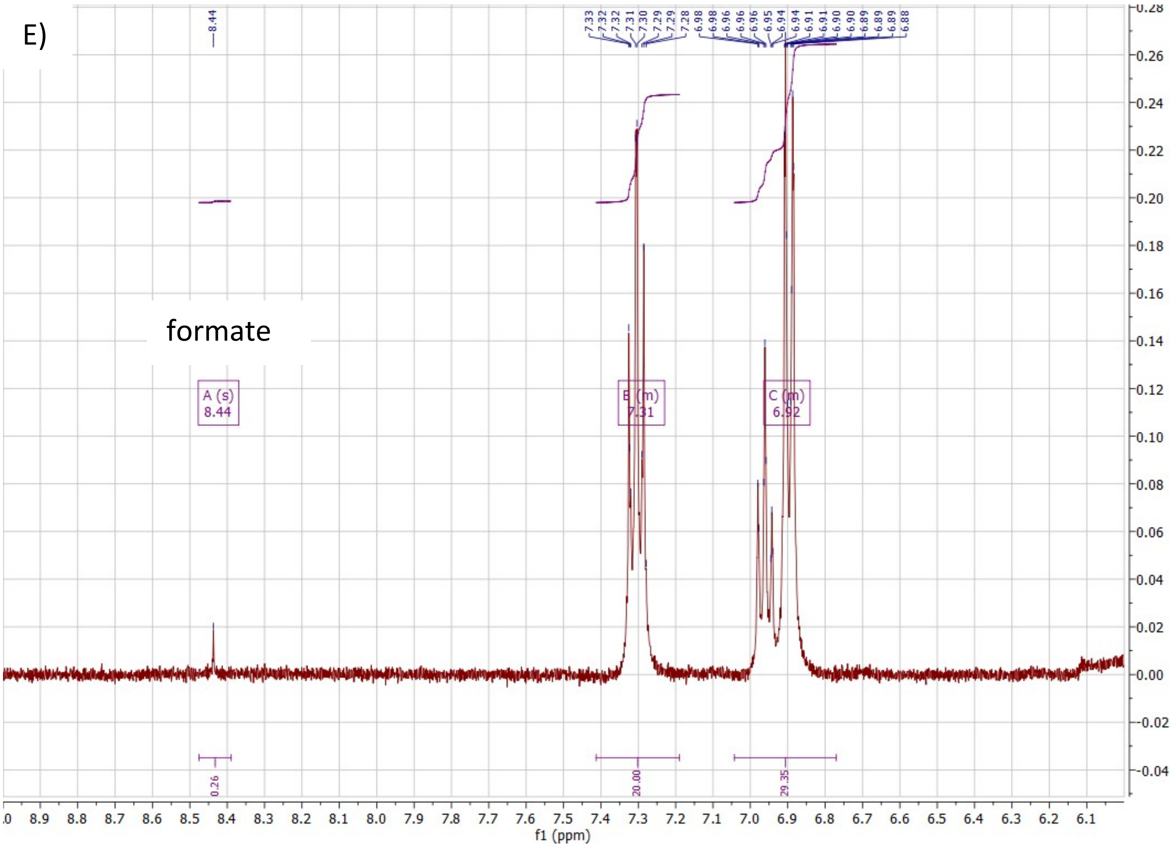

F)

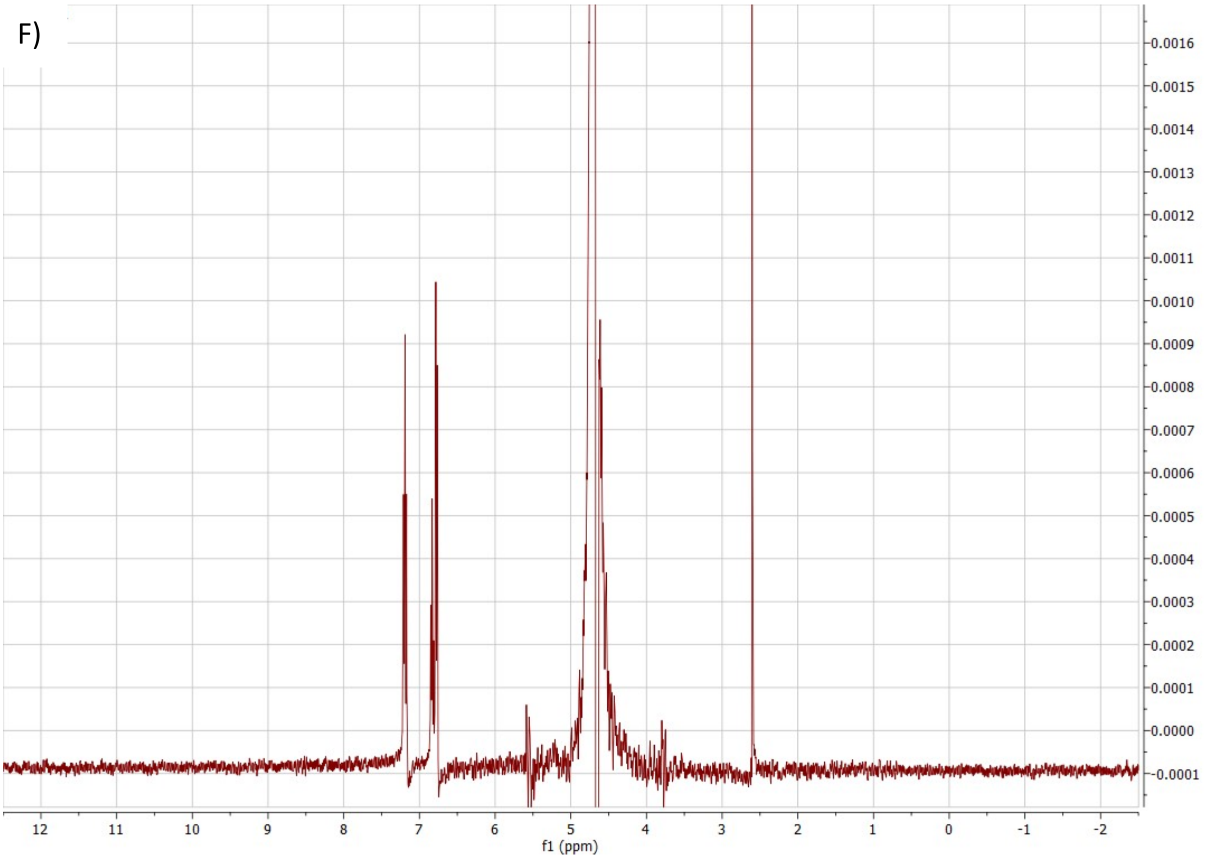

G)

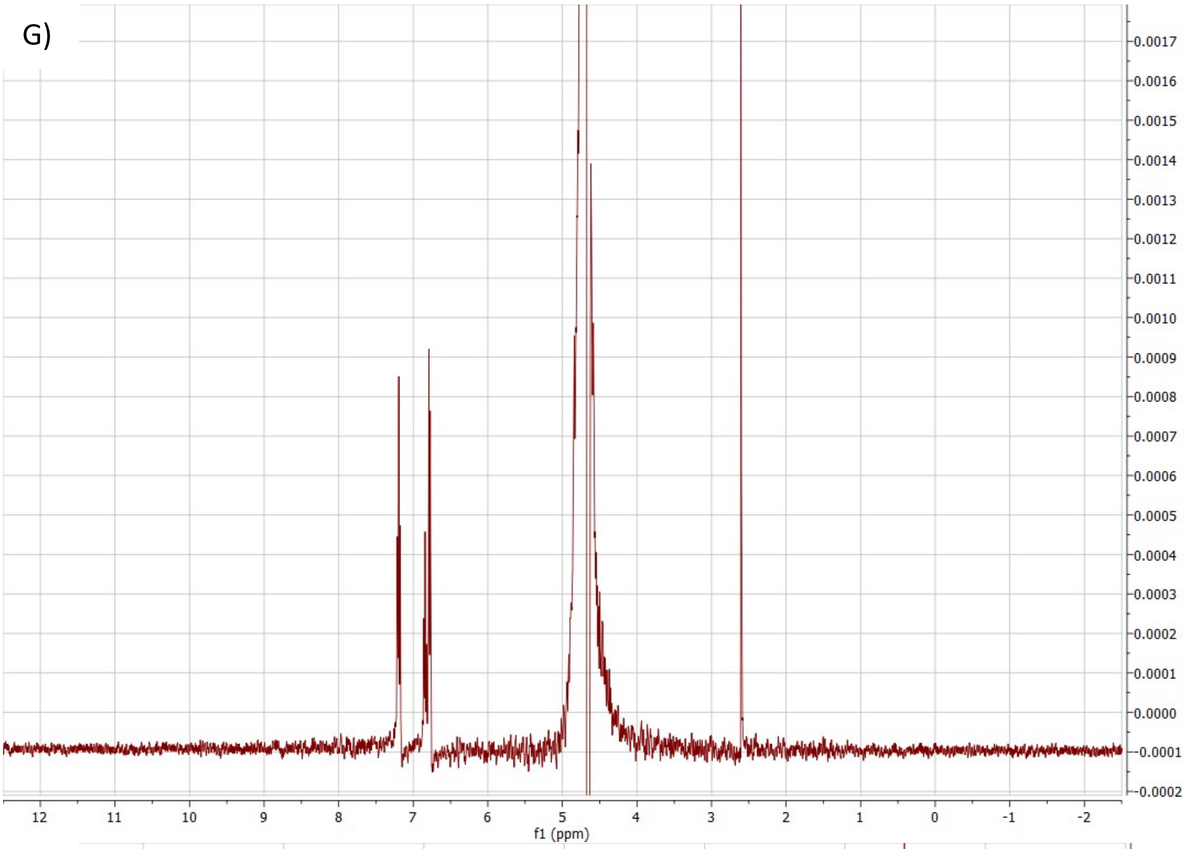

H)

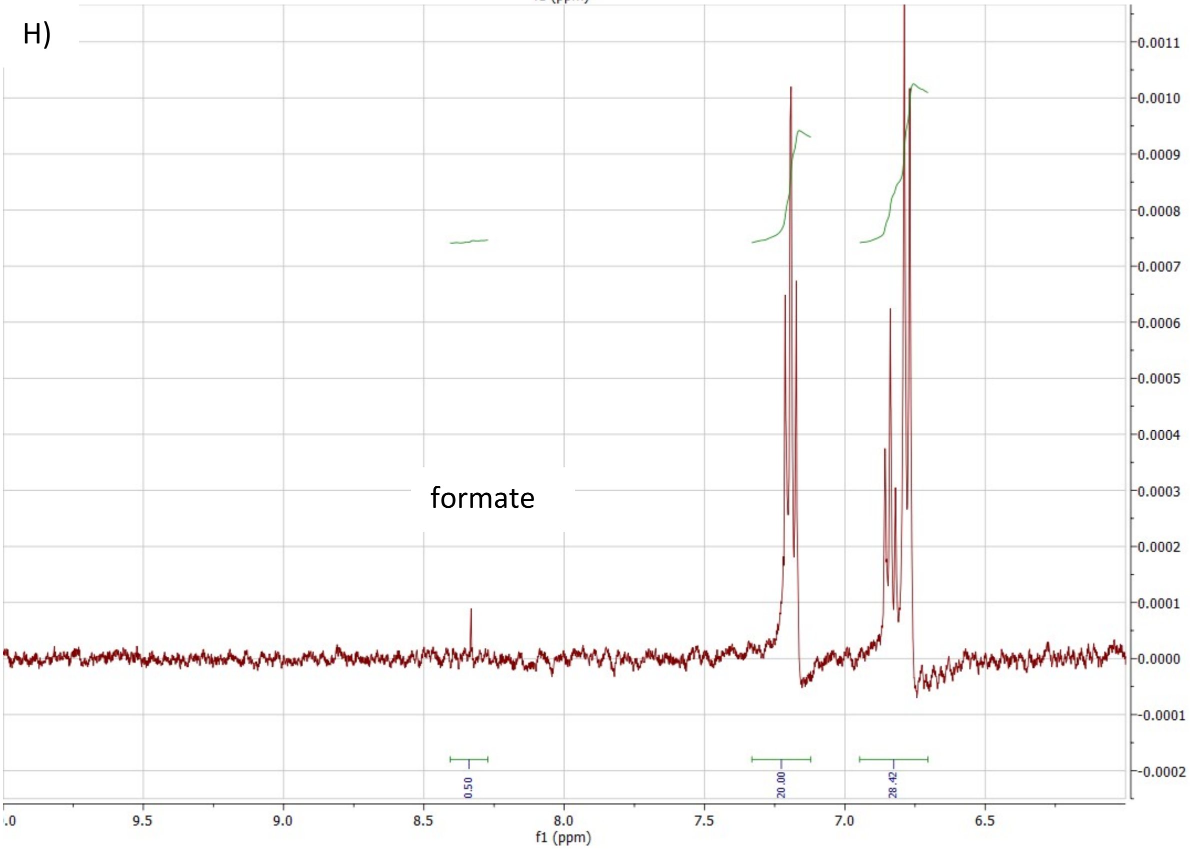

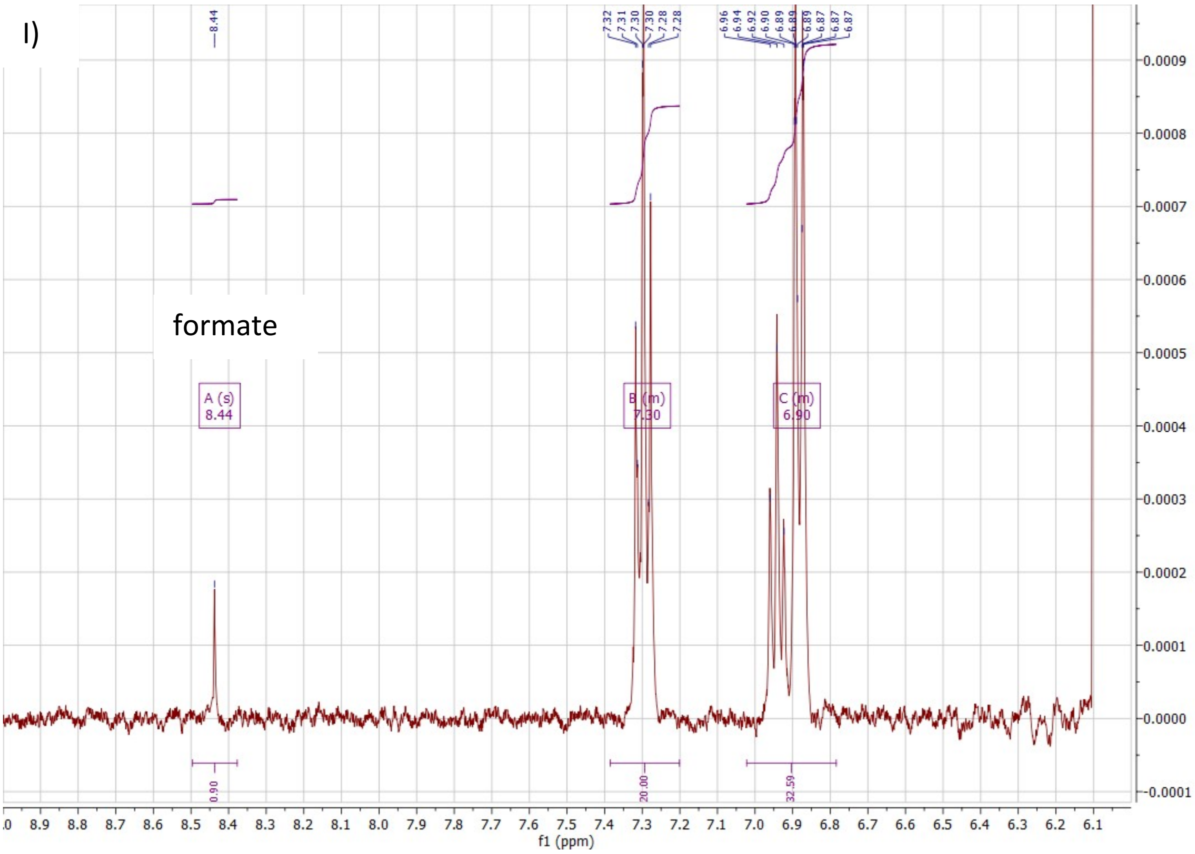

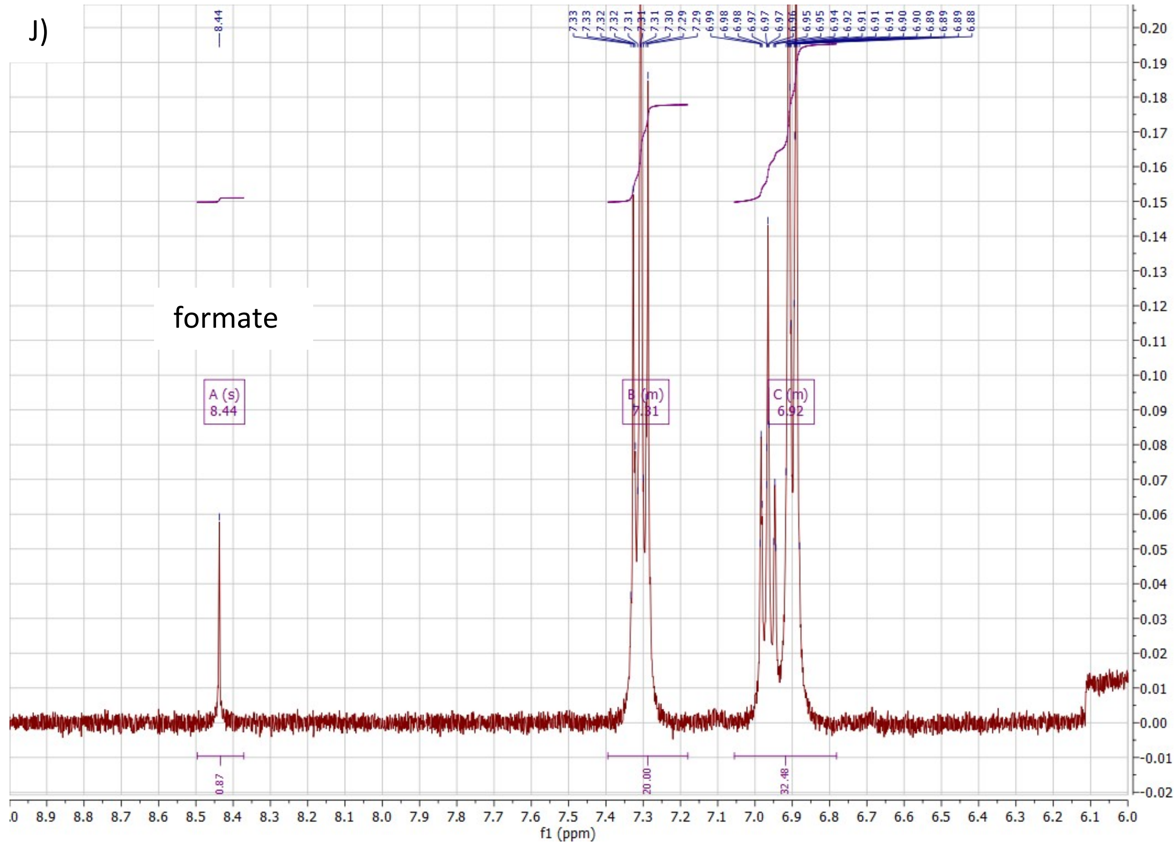

Figure S6: NMR spectra showing the (absence of) formate production for Ag-203 at a) -0.7 V vs RHE; b) -0.9 V vs RHE; c) -1.2 V vs RHE; d) -1.3 V vs RHE and e) -1.4 V vs RHE; and for Ag-372 at f) -0.7 V vs RHE; g) -0.9 V vs RHE; h) -1.2 V vs RHE; i) -1.3 V vs RHE; j) -1.4 V vs RHE;

F. Faradaic Efficiency

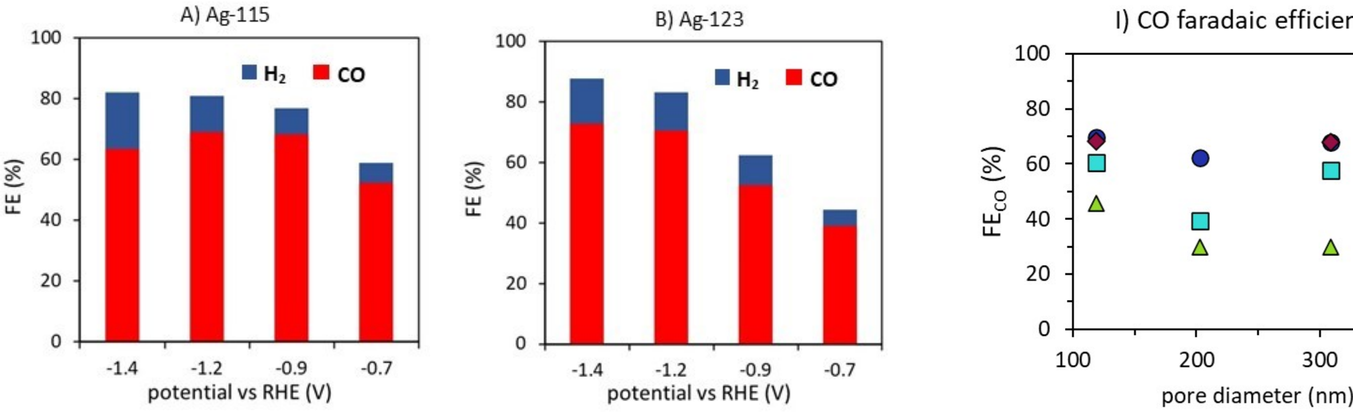

Figure S7: Faradaic efficiencies for the a) Ag-115, b) Ag-123, c,d) Ag-203, e,f) Ag-308 and g,h) Ag-372 catalysts when applying different potentials in catalysis and plots of the pore sizes dependence of the faradaic efficiency for i) CO and j) H_2 at different potentials

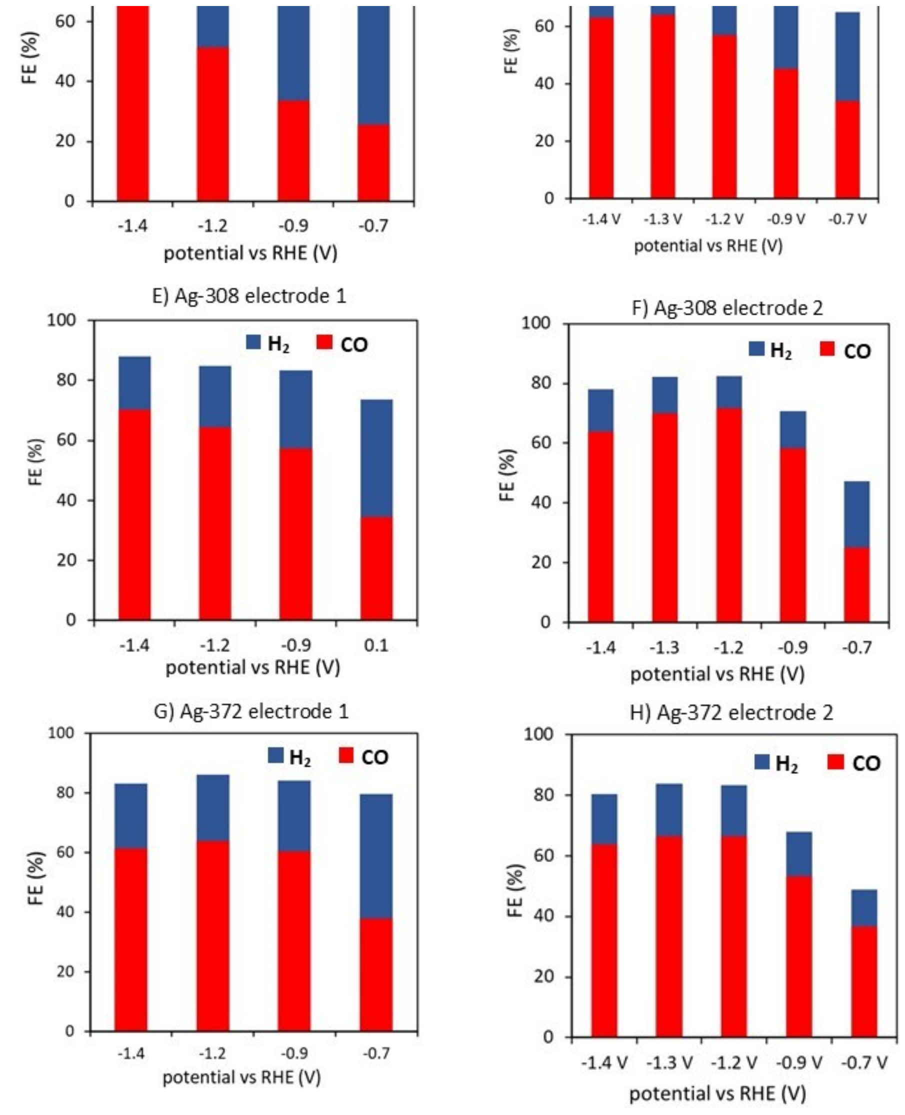

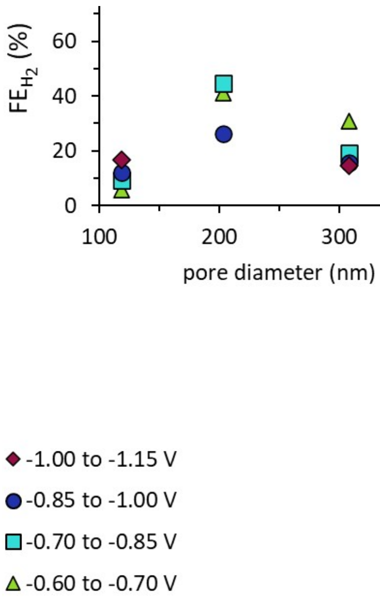

G. Sigmoidal fit catalytic data

All the data was fit with the Slogistic1 fit function in Origin, based on the function

$$y = \frac{a}{1 + e^{(-k(x - xc))}}$$

Table S2: R^2 values for the sigmoidal fits of the partial current density to CO and H_2 for the Ag-372, Ag-308, Ag-203, Ag-123 and Ag-115 catalyst.

Sample	*R^2 $\lvert J_{CO}\rvert$*	*R^2 $\lvert J_{H_2}\rvert$*
Ag-115	0.99600	0.99944
Ag-123	0.99964	0.99343
Ag-203	0.95932	0.50397
Ag-308	0.86573	0.42634
Ag-372	0.93996	0.95662

H. Surface area

Tabel S3: Pore diameter and ECSA determined via Pb UPD, DLC and EIS of the five porous Ag samples

Sample	*Ag pore size (nm)*	*ECSA (cm²/electrode) via Pb UPD*	*ECSA (cm²/electrode) via DLC*
Ag-115	115	67	139
Ag-123	123	65	112
Ag-203	203	33	93
Ag-308	308	18	45
Ag-372	372	19	84

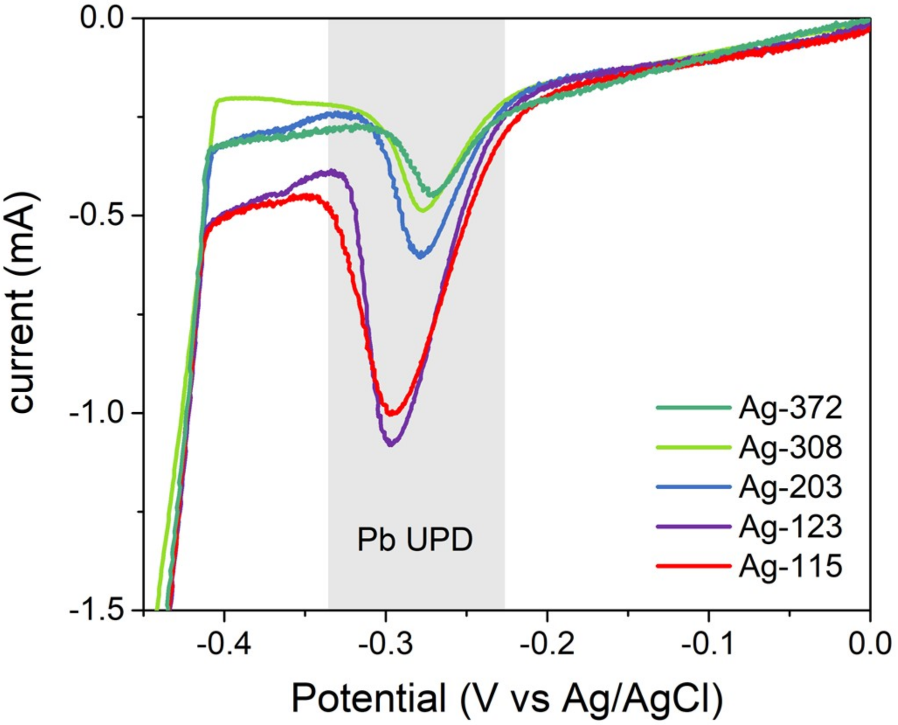

Figure S8: Pb underpotential deposition (UPD) curves for the Ag-115, Ag-123, Ag-203, Ag-308 and Ag-372 catalyst.

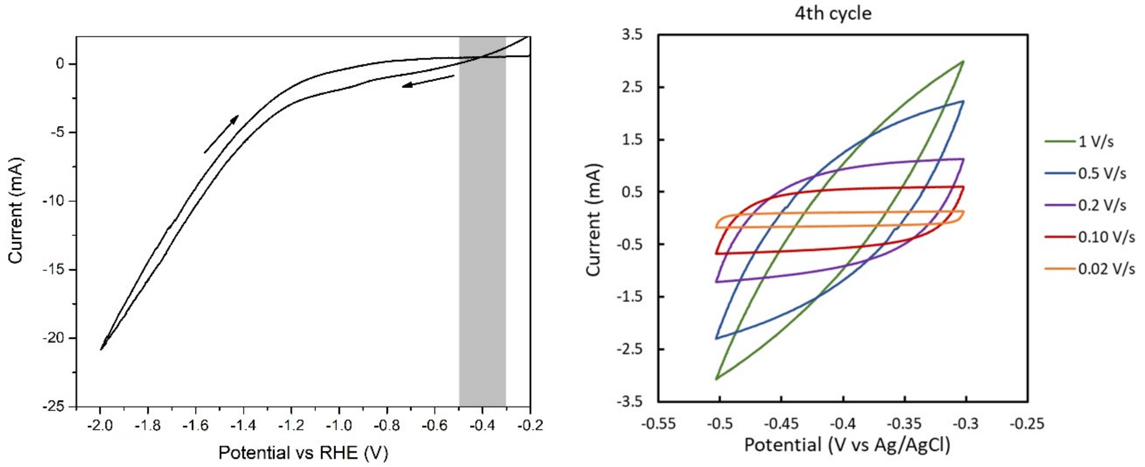

Figure S9: a) CV of a Ag-203 catalysts with 1 C/cm² Ag loading showing the non-linear regime used for DLCin grey; b) CVs obtained during DLC for a 2 C/cm² Ag-203 catalysts indicating that for larger scan rates (>0.2V), diffusion inside the porous Ag is not fast enough and the scans are no longer symmetric. These scan rates were excluded from the DLC determination.

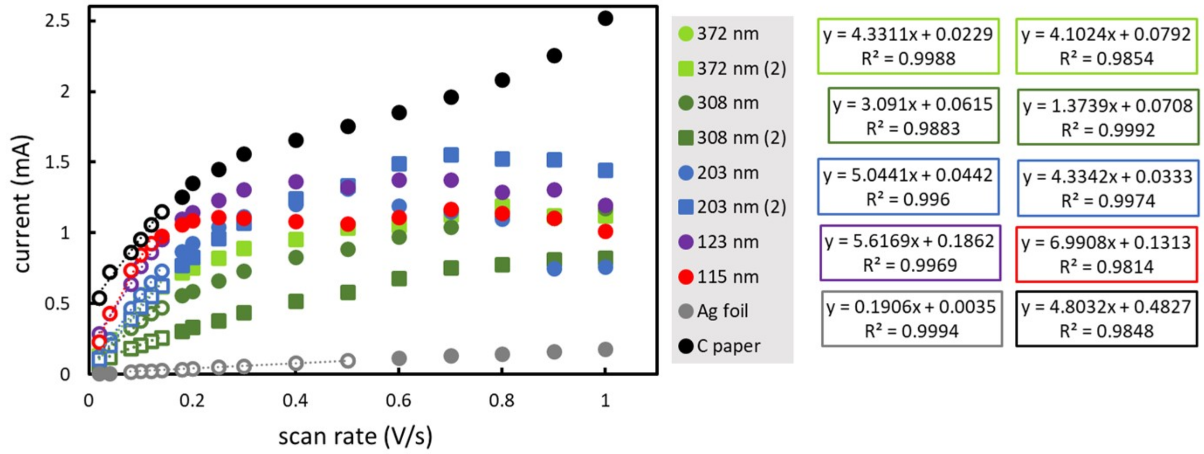

Figure S10: DLC curves for the Ag-115, Ag-123, Ag-203, Ag-308 and Ag-372 catalyst. The open data points indicate the linear parts that were used to determine the ECSA of the catalysts. On the right side, the linear fits (format ax + b) are given, where 'a' is the capacitance in mC/electrode.

I. FIB-SEM

Data analysis

The data analysis was performed in ImageJ. An image stack was created by selecting images that were not out of focus nor contained defects that were not representative for the whole sample. For Ag-115 the dataset was composed of 69 consecutive images ordered in the *z* direction, for Ag-203 two datasets of 91 images and 100 images were selected (of which the results were averaged after all analyses), and for Ag-372 100 images were included in the dataset. Then, the real voxel depth was determined based on the roundness of the voids and the StackReg plugin was used to align the stacks. (1) Next, the images were cropped to a common Region of Interest (ROI) in the image plane (x,y) of 3.00 µm x 2.00 µm. A Gaussian Blur filter with sigma radius 3 was applied to reduce the electron micrograph noise. Finally, the contrast in the images was enhanced by normalizing and equalizing the stack histogram. The different elements in the image (pore or Ag) were segmented *via* Trainable WEKA segmentation.(2) The default steps (Gaussian blur, Hessian, Sobel filter, difference of Gaussians and Membrane projections) were used with a filter sigma ranging from 1 to 16 and the FastRandomForest classifier algorithm. To overcome the anisotropy in the *z* direction (the voxel depth), the binarized slices were interpolated to create isotropic *x*, *y,* and *z* directions. The 3D Viewer plugin(3) of ImageJ was used to visualize and reconstruct the 3D surface from the segmented Z-Stack. A 3D watershed (3D ImageJ Suite)(4) was applied to separate the individual pores and by measuring the center-to-center distances of the pores,(4) the data for

the pore distance distributions was obtained. The pore volume was calculated by using the ratio between the segmented voxel of Ag and porous space. The Skeletonize3D plugin(5) of ImageJ was used to reduce the structure to its medial axis. The skeletons were then measured and characterized through the AnalyzeSkeleton plugin.(6,7) To avoid artefacts, the skeletonization analysis was performed with a prune cycle based on the shortest branch and the elimination of the prune ends. The Matlab App(8) TauFactor(9) was used to determine the diffusion in *x*, *y*, and *z* directions through the finite difference method and to determine the 3D pore volume.

The diffusional tortuosity was analyzed (based on simulations of the diffusion using $D_{eff} = p/\tau * D_{bulk}$, where τ is the tortuosity, D_{eff} the effective diffusion, D_{bulk} the diffusion in the bulk and p is the porosity) and not the geometric tortuosity (based on $\tau = R/L$, where R is the path through the pores and L the shortest path between begin and end), as the former accounts for the real characteristics of the diffusing substance.(10,11)

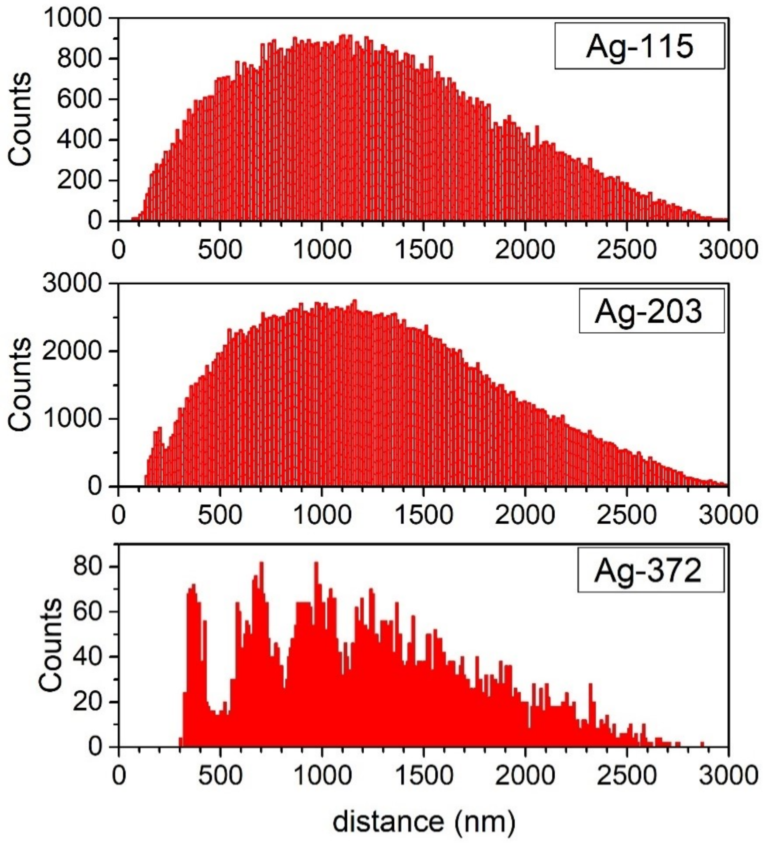

Figure S11: Distances distribution of the pore center to center distance for Ag-115, Ag-203 and Ag-372.

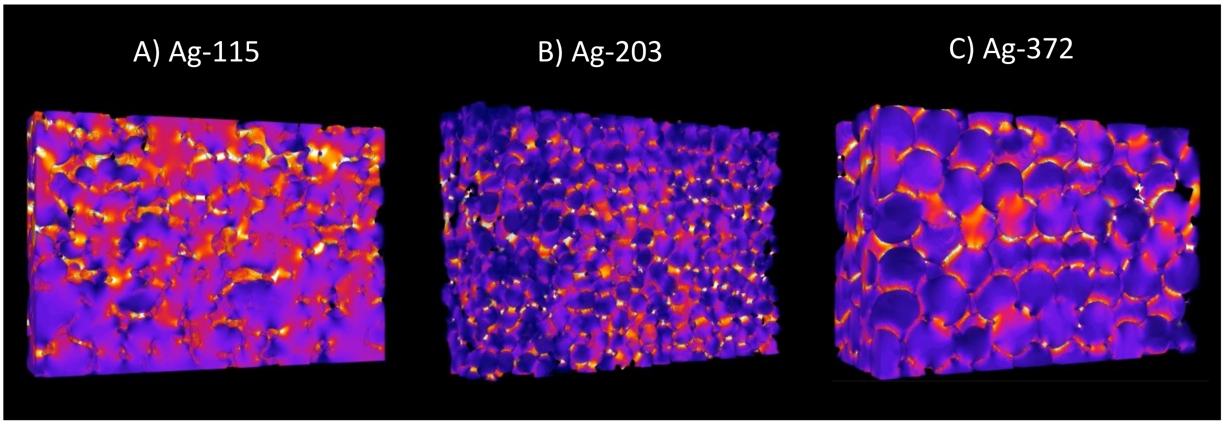

Figure S12: 3D diffusion model from the samples a) Ag-115; b) Ag-203; and c) Ag-372. The blue color indicates easy diffusion, the orange/yellow color harder diffusion.

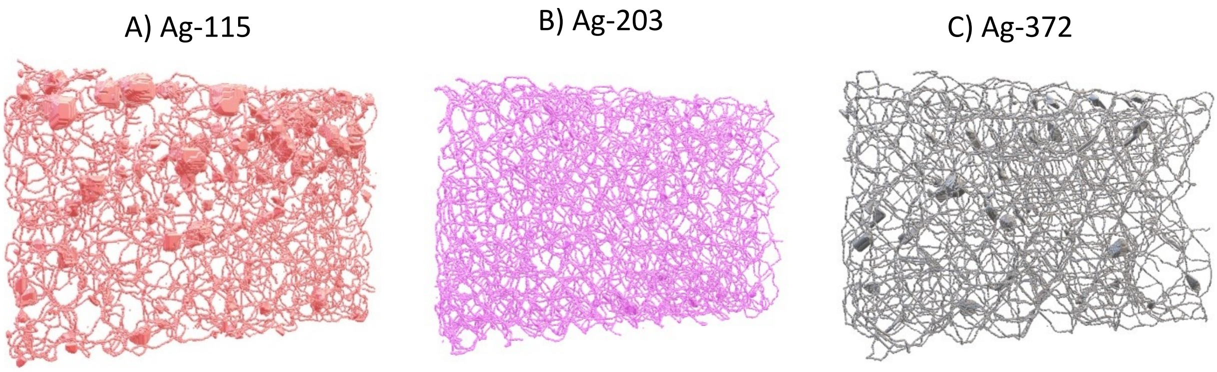

Figure S13: 3D skeleton based on FIB-SEM slice-and-view results from the samples a) Ag-115; b) Ag-203; and c) Ag-372

J. After catalysis

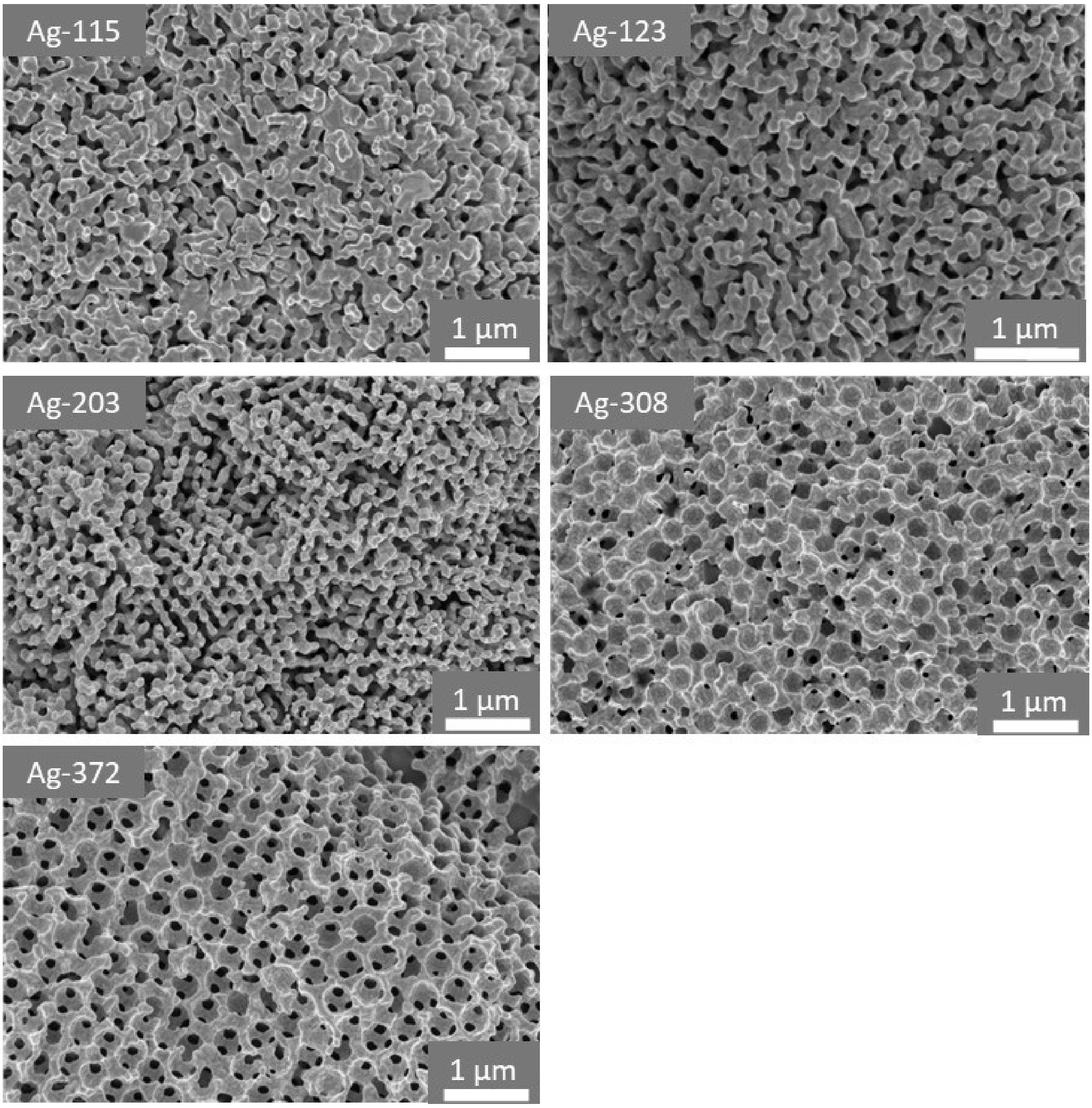

Figure S14: SEM images of the a) Ag-115, b) Ag-123, c) Ag-203, d) Ag-308 and e) Ag-372 samples after catalysis.

K. Long term catalytic performance

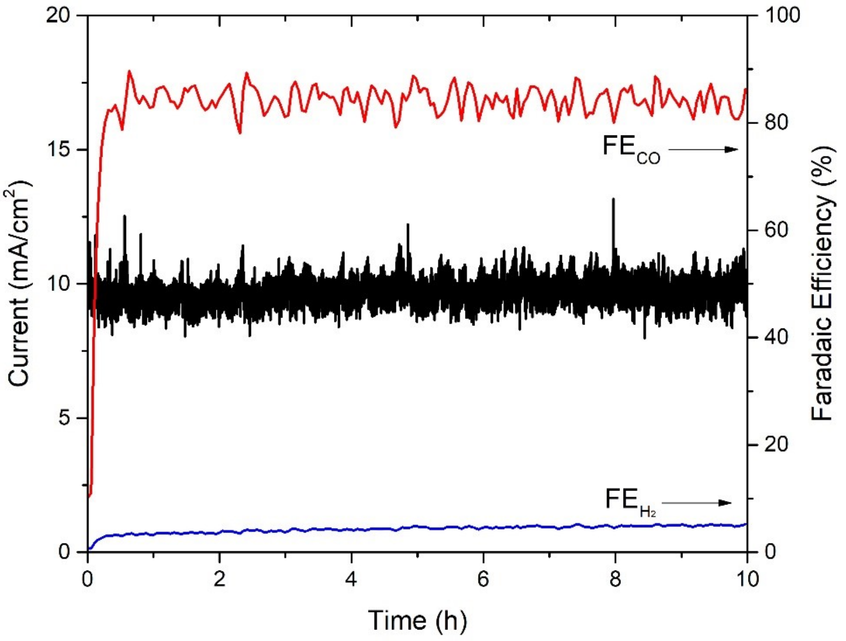

Figure S15: Catalytic performance over time of a Ag-145 catalyst with a Ag loading 3.3 mg/cm^2 on C-cloth at -1.2 V vs RHE (no iR correction). The catalytic performance was measured in duplo in a ElectroCell flow cell with a iridium oxide-based anode (Dioxide Materials) and an anion exchange membrane (Fumasep FAA-3-PK-130). A flow of 20 mL/min CO_2 was applied, as well as 15 ml/min electrolyte flow of 0.1 M $KHCO_3$. The long term stability was measured after applying a voltage of -0.7 V, -0.9 V, -1.2 V, -1.3 V and -1.4 V for 0.5 h each on a Autolab PGSTAT204.

References

1. Schindelin J, Arganda-Carreras I, Frise E, Kaynig V, Longair M, Pietzsch T, et al. Fiji: An open-source platform for biological-image analysis. Nat Methods. 2012;9(7):676–82.
2. Arganda-Carreras I, Kaynig V, Rueden C, Eliceiri KW, Schindelin J, Cardona A, et al. Trainable Weka Segmentation: A machine learning tool for microscopy pixel classification. Bioinformatics. 2017;33(15):2424–6.
3. Schmid B, Schindelin J, Cardona A, Longair M, Heisenberg M. Open Access SOFTWARE A high-level 3D visualization API for Java and ImageJ. BMC Bioinformatics. 2010;11:274.
4. Ollion J, Cochennec J, Loll F, Escudé C, Boudier T. TANGO: A generic tool for high-throughput 3D image analysis for studying nuclear organization. Bioinformatics. 2013;29(14):1840–1.

5. Lee T. Building Skeleton Models via 3-D Medial Surface/Axis Thinning Algorithms. Vol. 56, Graphical Models and Image Processing. 1994. p. 462–78.
6. Doube M, Klosowski MM, Arganda-Carreras I, Cordelières FP, Dougherty RP, Jackson JS, et al. BoneJ: Free and extensible bone image analysis in ImageJ. Bone. 2010;47(6):1076–9.
7. Arganda-Carreras I, Fernández-González R, Muñoz-Barrutia A, Ortiz-De-Solorzano C. 3D reconstruction of histological sections: Application to mammary gland tissue. Microsc Res Tech. 2010;73(11):1019–29.
8. MATLAB version: 9.13.0 (R2023a) [Internet]. 2023. The MathWorks Inc.
9. Cooper SJ, Bertei A, Shearing PR, Kilner JA, Brandon NP. TauFactor: An open-source application for calculating tortuosity factors from tomographic data. SoftwareX. 2016;5:203–10.
10. da Silva MTQS, do Rocio Cardoso M, Veronese CMP, Mazer W. Tortuosity: A brief review. Mater Today Proc. 2022;58:1344–9.
11. Fu J, Thomas HR, Li C. Tortuosity of porous media: Image analysis and physical simulation. Earth Sci Rev. 2021;212(October 2020):103439.
